# Mental Representations of Impossible Non-Euclidean Environments

**DOI:** 10.1007/s00426-026-02297-3

**Published:** 2026-05-13

**Authors:** Andrew S. McAvan, Timothy P. McNamara

**Affiliations:** Department of Psychology, Vanderbilt University, Nashville, TN USA

Computer scientists and frequent programmers often use “defensive design” philosophy to create robust software, often with the purpose of “breaking” a program to effectively reveal unintended and sometimes devastating edge cases. Imagine if someone inputs a string of code designed to delete an entire database into an unsecured password field. Known as an “injection attack,” this exploits an edge case within the database program’s code, allowing a malicious actor to delete important data, highlighting just how critical edge cases can become.

What if we were to apply this concept to test the brain for edge cases? Naturally, we cannot manipulate real-world paradigms to create experiments that that violate universal laws. However, we can use virtual reality (VR) to construct novel environments that violate fundamental aspects of our three-dimensional experience. By observing how the brain reacts to these impossible edge cases, we can reveal cognitive behaviors without the influence of any prior experience or knowledge.

Although the surface of the Earth is described intrinsically by spherical geometry (i.e., on the positively curved surface of a sphere), the local physical environment—the environment of direct human experience—can be approximated by Euclidean geometry (as described in Euclid’s, Elements (300/2017). Interestingly, the shape of the universe seems to align with Euclidean geometry (De Bernardis, et al., 2000; Bennett, et al., 2013; Ade, et al., 2014). Euclidean geometry is a good approximation of the spaces that humans have navigated by foot, pack animal, and terrestrial vehicle for hundreds of thousands of years (Auslander & Kuranishi, 1957; Gauld, 1974; Lee, 2010).

Spaces that violate the axioms of Euclidean geometry have been represented across popular culture. Films such as *The Cabinet of Dr. Caligari* (1920), prints like M.C. Esher’s *Relativity* (1953), videogames like the *Portal* series (2007 & 2011), and the long-running television series *Dr. Who* (1963—present) all exemplify some form of manipulation that violates Euclidean geometry. However, research on how humans represent navigational spaces containing these manipulations is in its early stages (e.g., Warren et al., 2017). We use the term “non-Euclidean” to refer to spaces that produce inconsistencies with the local spaces of human experience and navigation. These spaces may violate one or more axioms of Euclidean geometry (e.g., spherical spaces) or may be impossible to experience directly (e.g., a 4-dimensional environment).

Research on spatial learning and navigation in non-Euclidean environments is important because it may be informative about the nature of spatial representations in the brain and whether those representations are better characterized as cognitive maps (e.g., O’Keefe & Nadel, 1978) or as cognitive graphs (e.g., Chrastil & Warren, 2014). A cognitive map represents straight-line distances and directions between locations using a globally consistent coordinate system (O’Keefe & Nadel, 1978). Cognitive maps may be distorted and inaccurate (e.g., Tversky, 1981) but they adhere to the principle that the spatial layout of an environment is represented in an internally consistent manner. In cognitive graphs, locations are represented by nodes and the paths between them are represented by edges. The representation can contain local, quasi-metric information (e.g., the turn is about 90 degrees, the distance from A to B is about 50 m) but it can use different frames of reference in different locales (e.g., “north” in one place might not be aligned with “north” in another place) and permits geometric inconsistencies (e.g., two “90 degree” turns lead back a starting location). One can think of a cognitive graph as a generalized and less precise, albeit more flexible, cognitive map, with the graph lacking the full survey knowledge and global scale of the map.

One vein of research on the learning of non-Euclidean environments has explored “wormholes,” or distortions which transport a participant from one location to another location discontinuously; in effect, the distance between two distinct locations is reduced to zero at the wormhole. Warren et al. (2017) tested participants in a fully immersive VR maze environment that had various locations connected via wormholes. The goal was to determine whether and to what extent humans could incorporate non-Euclidean information into their underlying spatial representations. Different from traditional Euclidean shortcuts that allow for optimal traversal through an environment, Warren et al.’s wormholes essentially shrank and rotated opposing quadrants of the virtual space. When participants encountered these distortions, they did not notice the distortions during travel, despite using the routes within the maze just as effectively as their Euclidean control counterparts. With fewer errors and shorter overall routes, participants demonstrated the ability to effectively acquire path knowledge in a non-Euclidean context. Furthermore, when performing a shortcutting task, participants’ memories for object locations were heavily biased by the locations of the wormholes, and when sketching maps from memory, participants recreated environments in ways that reversed and swapped the orders of objects via a hypothesized series of “stretches, rips, and folds” (Warren et al., 2017). Warren et al. concluded that the behavior of navigators in these worm-hole environments was more consistent with cognitive graphs than cognitive maps (see also, Ericson & Warren, 2020).

Muryy and Glennerster (2021) explored route selection across fixed (zero-wormhole), one-wormhole, and three-wormhole environments and found that participants’ choice selection strategies differed between the Euclidean and non-Euclidean environments. In the Euclidean maze environment, participants’ choices at various junctions were best predicted by a shortest-distance model, whereas participants’ choices in the non-Euclidean environments were best predicted by a rewarded-choice model. Essentially, when in a simpler environment (i.e., one with fewer wormholes), people opted to use a place-based strategy and in more complex environments people tended to use a response-based strategy (e.g., Shelton et al., 2013). However, differential strategy usage was found to be driven by the overall complexity of the environment: Participants in the three-wormhole environment relied on previously remembered choices more than those in the one-wormhole environments (Muryy & Glennerster, 2021).

Other researchers, however, have suggested that these wormhole environments, while non-Euclidean, still allow for participants to stretch, rip, and fold the environment in ways that manage to preserve certain Euclidean aspects in their underlying spatial representations (Widdowson & Wang, 2023). One could even argue that wormhole traversal operates in a manner like teleportation; they both instantaneously move an agent through space by effectively reducing the distance between two disparate locations to zero. Interestingly, not only is teleportation seen across numerous media (such as the *Portal* video game series), but it is also one of the most common locomotion modalities utilized within contemporary VR programs (Boletsis, 2017; Boletsis & Cedergren, 2019).

Aside from teleporting wormholes, other non-Euclidean manipulations have been explored and found to bias people to a possible Euclidean representation of an impossible non-Euclidean space. For example, Du et al. (2023) had participants learn, in a virtual reality hallway, a route that should have intersected itself based on the lengths of segments and angles of turns of the outbound route, but no such intersection was visible to participants (as if the route passed over or under itself). They found that when tasked with pointing back to a starting location after walking these halls, participants tended to point in ways that more closely aligned with a representation that stretched or shrank parts of the hallway to better match a non-overlapping Euclidean representation (Du et al., 2023).

Widdowson and Wang (2022) and Kim and Doeller (2024) utilized curved geometries (hyperbolic & spherical, and spherical, respectively). Both experiments had participants virtually navigate these non-Euclidean spaces to find and remember the locations of specific objects and then point back to the locations. They found that even when knowledgeable about the curvature of the explored space, participants often pointed in ways that more closely resembled the spaces if they were flattened to match a Euclidean plane (Kim & Doeller, 2024; Widdowson & Wang, 2022). Taking it a step further, Kim and Doeller also had participants physically walk back to the remembered locations of objects and found further evidence that participants’ responses more closely aligned with a flattened Euclidean-plane-like representation instead of one representing the actual curved space.

One less-researched non-Euclidean distortion is the overlapping, superimposed space (e.g., the *Dr. Who* TARDIS being “larger on the inside”). Studies that examined the efficacy of these spaces in virtual architecture largely focused on whether naïve participants noticed the manipulation (Robb & Barwulor, 2019; Suma et al., 2012). These studies found that spatial overlap went widely unnoticed (Robb & Barwulor, 2019; Suma et al., 2012), especially when the experiments incorporated line-of-sight cameras aimed at increasing cognitive load (in the form of active detection avoidance; Ciumedean et al., 2020). However, the participants’ corresponding cognitive representations remained largely untested because the researchers only hypothesized about the conspicuousness of the space and not how the space was explored and represented.

The current project also investigated spatial learning and navigation in overlapping spaces. Participants walked between two side-by-side virtual rooms that unbeknownst to them overlapped in the center (Fig. 1). After experiencing this non-Euclidean environment, participants completed pointing and map-building spatial tasks. Performance in these tasks was used to assess how the spaces were mentally represented. If participants in the non-Euclidean environment could accurately perceive and encode the impossibly overlapping space without reverting to their Euclidean heuristics, then they should accurately point to the locations of target objects. Furthermore, participants would construct maps of the environment that demonstrated the central overlap of the two rooms. However, if participants were unable to reconcile the conflicting non-Euclidean information and instead reverted to their heuristic knowledge of Euclidean space, then they should inaccurately point to objects based on where Euclidean heuristics would dictate the objects’ locations to be instead of where the objects were located. Participants would also construct maps that are more like the Euclidean layout they *did not* experience than the non-Euclidean layout they *did* experience (e.g., leaving out the central overlap in-favor of non-overlapping neighboring rooms).

**Fig. 1:**
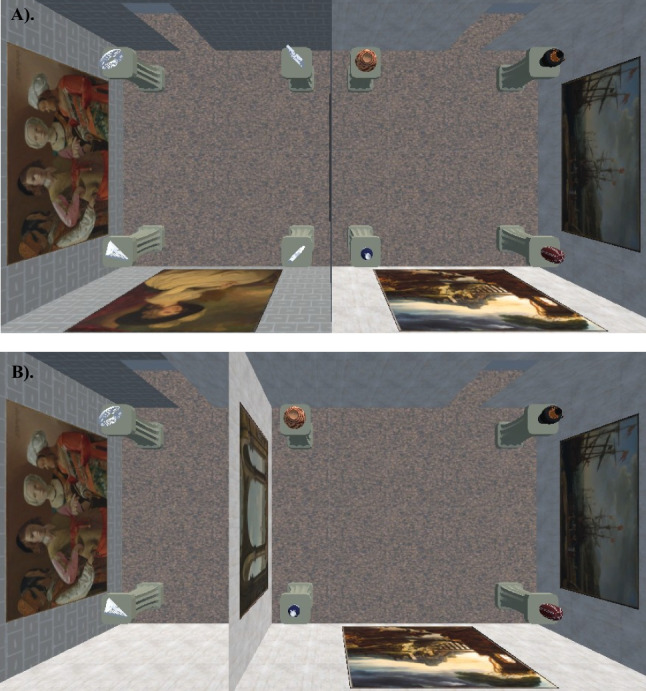

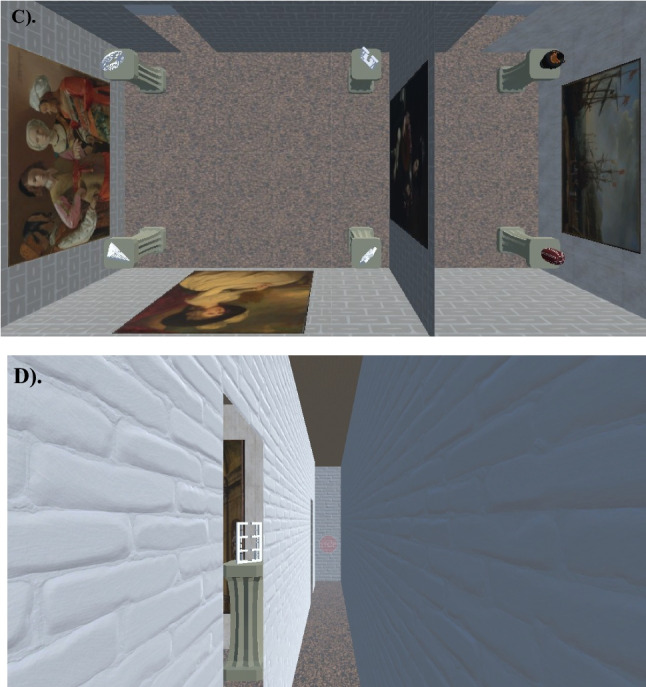
Overhead view of virtual rooms. a). Euclidean sub-rooms b). non-Euclidean sub-rooms with the “Maze” room demonstrating the overlap. c). non-Euclidean sub-rooms with the “Vase” room demonstrating the overlap. d). first-person view of the adjoining hallway w/ stop sign present

## Method

### Participants

We recruited 74 (37 female) undergraduate and graduate students from Vanderbilt University and the surrounding area to be participants. Ten participants (5 female) were dropped from the final sample due to not finishing on time (2) or to unforeseen computer errors that forced them to end the experiment early (8). No participants dropped out due to cybersickness or reported symptoms before, during, or after the experiment. Our final sample was 64 participants (32 female) whose ages ranged from 18 to 31 with a mean age of 21.89 (*SD* = 3.79). Upon signing up for the study, participants were assigned to eight between-participant conditions: sex (male or female), type of environment (Euclidean or Non-Euclidean), and testing order (map-making task followed by JRD task or vice versa). A priori power analyses with an effect size *f* = 0.75, α = 0.05, and Power (1 – β) = 0.95 in G*Power 3.1.9.7 (Faul et al., 2009) suggested sample sizes of 6 per group (47 total), but for more robust pairwise comparisons, we increased the sample size to 8 (64 total). Participants either received course credit for their participation or were monetarily compensated with an electronic gift card. All participants reported having normal or corrected-to-normal color vision and normal or corrected-to-normal visual acuity. Written informed consent and demographic information were obtained prior to the experiment, and the methods were approved by the Institutional Review Board at Vanderbilt University (#221,064).

### Materials

**Fig. 2:**
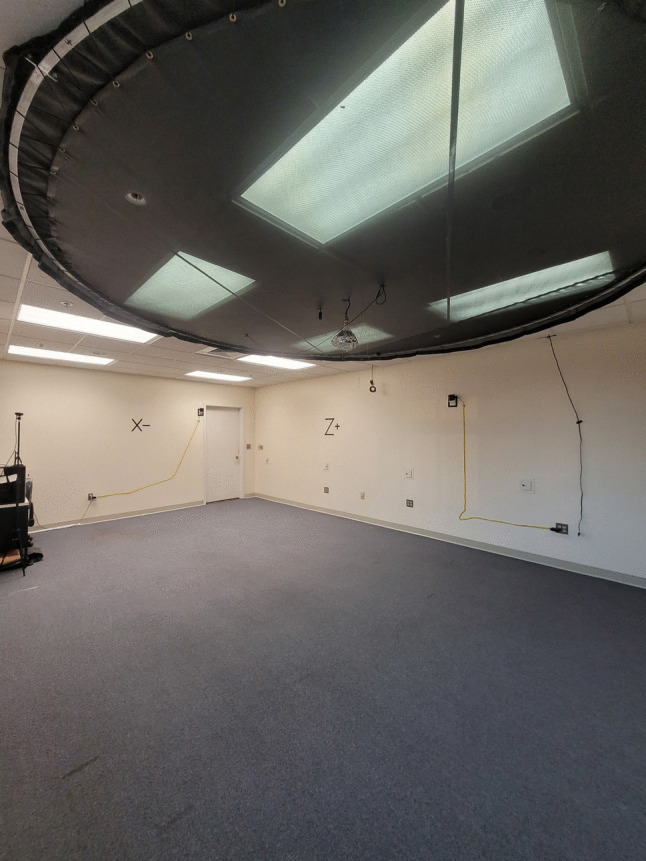
First person perspective of the real-world navigation space

**Fig. 3:**
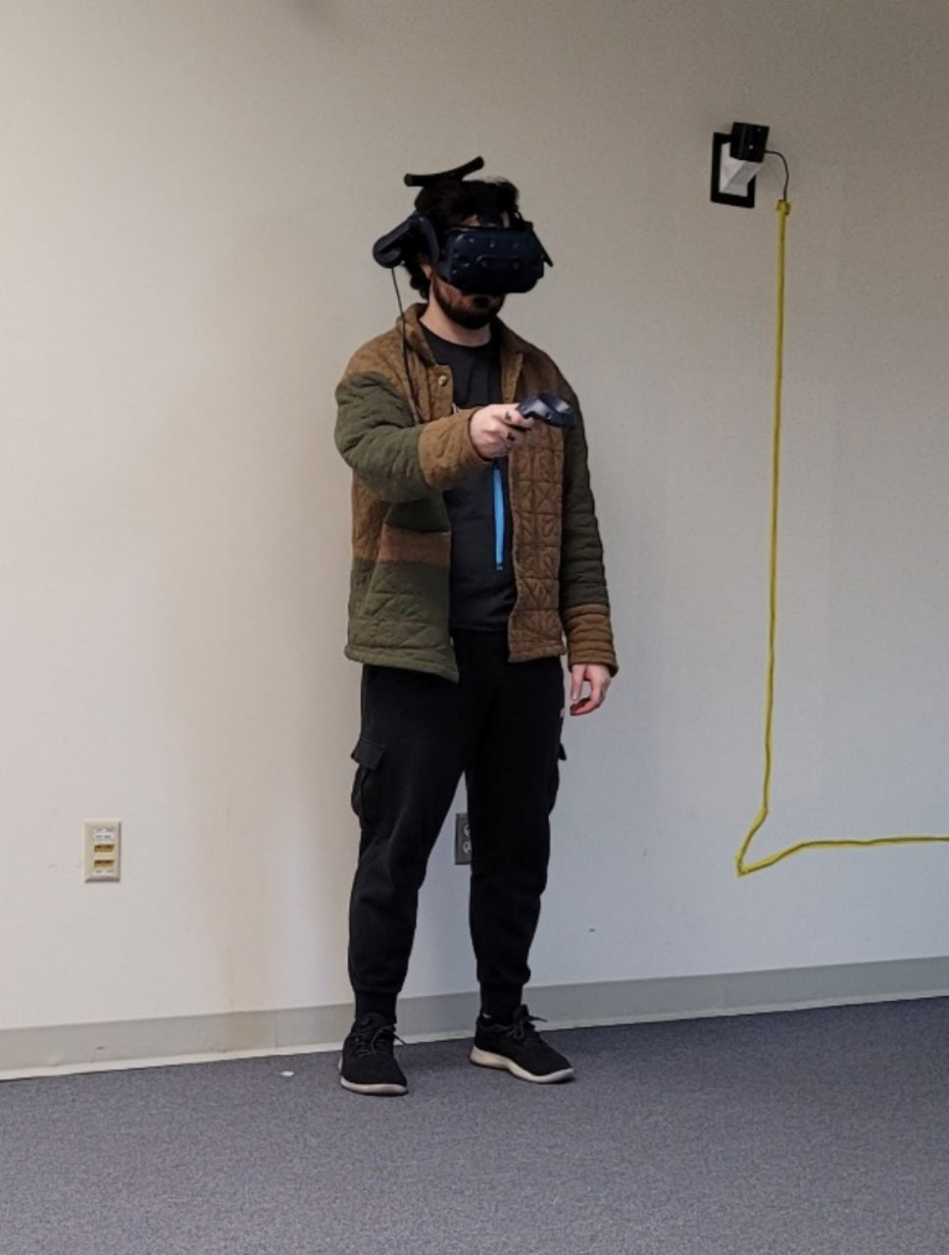
Researcher donning the immersive virtual reality setup (Vive Pro 1 HMD w/ Wireless Adapter, battery pack, and controller)

The virtual environment and experimental tasks were built in the Unity 3D (Unity Technologies ApS, San Francisco, CA) and utilized the Landmarks 2.0 Virtual Reality Navigation Package (Starrett et al., 2020). The walkable environment was approximately 5 m by 7 m in size (Figs. 1 and 2), with a non-navigable hallway extending ~ 1 m beyond the bounds of the room on three sides. This was done to give a sense of continuity to the space, but for safety, slightly transparent stop signs (~ 22% opacity) were rendered so that participants could see the invisible bounds. The space was divided into two sub-rooms of equal size with a connecting hallway. Some virtually rendered walls matched the positions of the physical room walls and others did not; before donning the VR equipment (Fig. 3), participants were instructed to treat all walls as if they were physically real and were specifically told not to “stick any part of their body through the walls or attempt to walk through any of the walls.” Each room contained a set of 4 unique target objects, for a total of 8, that were differentiated by at least one salient physical property: the maze objects were differentiated by shape (e.g., rectangular, circular, triangular, and star-shaped) and the vase objects were differentiated by their primary color (e.g., red, black, blue, and tan). All objects were placed atop pedestals of equal dimensions. Each sub-room contained a set of three unique paintings (sourced via the New York MET’s Open Access Initiative online assets; see Supplemental Materials), for a total of 6, to serve as proximal landmarks and aid with spatial memory. The “Maze Room” contained paintings depicting various portraits and the “Vase Room” contained paintings depicting various landscapes. The walls of each room and adjoining hallway were textured with different patterns so that each area was distinct and provided a sense of optic flow. The ceilings and floors each used a single texture (all textures sourced via Poliigon’s free online assets; see Supplemental Materials).

To allow for the full range of body-based cues provided by fully immersive, untethered VR we used the HTC Vive Pro 1 head-mounted display (HMD) along with the HTC Wireless Adapter (HTC, New Taipei City, Taiwan). The HMD displayed stimuli at a resolution of 1,140 × 1600 pixels per eye with a 110-degree field of view at a rate of 90 Hz. To allow the participant to fully ambulate through the VR environment without being connected via cable to a computer, we used the HMD in conjunction with the Wireless Adapter (HTC, New Taipei City, Taiwan). This allowed for uninterrupted exploration while delivering data from anywhere in the room. To record responses and allow participants to interact with the virtual environment, we used a single handheld HTC Vive Pro controller (HTC, New Taipei City, Taiwan). To record positional and response data, we tracked the HMD and controller using four version 2.0 Steam VR Base Stations (Valve Corp., Bellevue, WA, United States). The tasks were run on an Origin High-Performance PC with an NVIDIA RTX 2070 graphics card (Origin PC, Miami, FL, United States; NVIDIA Corp., Santa Clara, CA, United States).

### Procedure

After reading and signing the consent form, participants completed a set of demographics questionnaires. These included the Santa Barbara Sense of Direction Scale (SBSOD; Hegarty et al., 2002) to assess spatial skills, the Virtual Reality Sickness Questionnaire (VRSQ; Kim et al., 2018) to assess propensity for cybersickness, a videogame usage questionnaire to assess how often and how long participants would engage in playing videogames (in hours per month), and a general technology familiarity scale to assess for how well they know/use technology. If participants were receiving financial compensation, they also filled out a compensation form prior to start. After completing all the relevant paperwork, participants were led into the real-world navigation space. The procedures for the immersive VR portion of the experiment were verbally explained before participants donned the wireless HMD, controller, and clip-on battery pack (with the help of a researcher if necessary). Using the first visual instruction screen seen in Unity, the inter-pupillary distance within the HMD was adjusted until the text was as clear as possible for each participant.

## Design

### Euclidean and non-euclidean layouts

Participants were randomly assigned to one of two experimental conditions: In the Euclidean condition, participants experienced a virtual environment that corresponded to the normal physical world; in the non-Euclidean condition, participants experienced an environment that conflicted with basic geometric properties of space; in particular, the internal dimensions of the sub-rooms were larger than their external dimensions. The internal dimensions for the non-Euclidean sub-rooms were 4.1 × 3.75 × 2 m per room (61.5m^3^ total with a 1 m central overlap), whereas the external measurements of the rooms were 7.2 × 3.75 × 2 m (54 m^3^ total), creating a navigable space that was larger on the inside than the outside. The internal dimensions for each Euclidean sub-room was 3.6 × 3.75 × 2 m (54 m^3^ total), matching their external measurements.

### Immersive virtual navigation tasks

All participants received the same paradigm in the same order: a five-minute-long exploration phase, followed by eight trials of a visible target learning phase, and finally eight more trials of a non-visual target probe phase. Before every trial, they experienced a reorienting procedure in which the entire environment disappeared, leaving only a red visible start location that randomly changed its position along the adjoining hallway every time. Participants were instructed to approach the red visible start object (circle with an arrow) from the front, match its facing direction until it changes color (to green), and then press a button on their controller to mark that they were ready to begin. For participant safety, the start object had a directional arrow pointing away from the physical wall, and participants were told that the starting locations were along a wall and facing away from it. In total, each participant experienced 17 total trials and 17 reorienting procedures within VR.

### 5-Minute exploration

The exploration task served a dual purpose: to familiarize participants with the environment and target objects within it, while also allowing them to get used to walking in VR. Before the exploration trial, participants received a set of visual instructions that matched the previously given verbal instructions. After reading and understanding the instructions, the participants started exploring for five minutes. During this time, all the target objects were visible within the rooms and participants simply had to walk around the space and encode it. All participants were instructed to explore for the five-minute duration and experienced each room and the adjoining hallway at least once. After the set time was up, participants immediately moved on to the next portion.

### Visible target learning trials

After the exploration phase, participants completed a single block of eight navigation trials wherein they were tasked with navigating to a given visible target object. After completing the reorienting procedure, participants were visually prompted via text to find one of the eight target objects (e.g., “Your target is the Black Vase”). The target objects were presented randomly and without repetition so that each participant navigated to each target object in a random sequence. All eight target objects were visible from the start, and participants had to simply walk over to it and interact with the salient object by physically putting their controller through it to move on. The purpose of this task was to help participants further learn the locations of the target objects in the virtual environment as well as familiarize them with how the target objects would be referenced in future portions of the study.

### Invisible target probe trials

Following the learning phase, participants then completed a single block of eight navigation trials wherein they were tasked with navigating to the remembered location of an invisible target object. After the reorienting procedure, participants were prompted to find one of the eight target objects (in the same way as the previous block). The target objects were again presented randomly and without repetition so that each participant’s memory for the target objects was probed in a random sequence. All target objects (and their pedestals) were invisible from the start, and participants were tasked with walking to the remembered location of the prompted target and pressing a button on their controller to mark said location as their response. When their response was recorded, the distance error between their response location and the central location of the target was calculated and recorded as well.

None of the target objects ever changed virtual locations or rotations and were the same for all participants.

### Non-immersive virtual tests

Upon completing the immersive VR portion of the study, participants then doffed their VR gear with the assistance of a researcher and were ushered to a desktop computer to begin the non-immersive desktop VR testing phase. Participants received the testing paradigms in order based on their previously determined groups: Map-making first or Judgements of Relative Directions first. Prior to each task, participants received a set of verbal instructions that matched the visually presented instructions on screen.

### Map-making task

**Fig. 4:**
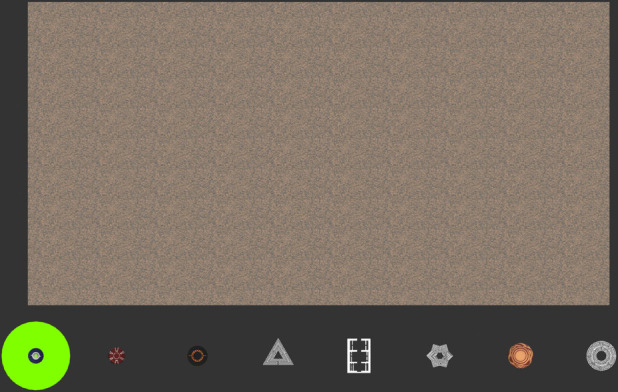
Example of the desktop map-making task

Participants were tasked with recreating a single overhead map of the two sub-rooms they just experienced. A top-down view of the floor, without any walls, as well as top-down views of the vase targets were rendered (Fig. 4). The maze targets were rendered as having their forward directions facing upward (toward to participant) as they were distinguished by the shape of their forward (and backward) faces while their overhead and side profiles were practically indistinguishable. All the target objects started out below the mappable area in a randomized order, with the first in the sequence being highlighted by a green circle. Participants could switch between the targets at any time using the comma and period keys (or left- and right-angle bracket keys) with the newly switched object being highlighted. To move the objects to their remembered locations within the mappable area, participants pressed the arrow keys to move the highlighted object in the related direction. For example, pressing the up-arrow key would move the object upward on the screen. Participants were allowed to press and hold the shift-key to double the movement speed of the object to allow for quicker traversal across the map area. Once all the objects were mapped to their remembered locations and participants were satisfied with their choices, they submitted their map data by pressing the enter key. Data for the target objects were stored in cartesian coordinates. If participants accidentally entered their map data before they meant to, they simply had to inform a researcher who would restart the map-making task. Participants were informed that there was no time limit and to try to be as accurate as possible. These instructions were given to remove any time pressure and increase map reliability.

### JRD task

**Fig. 5:**
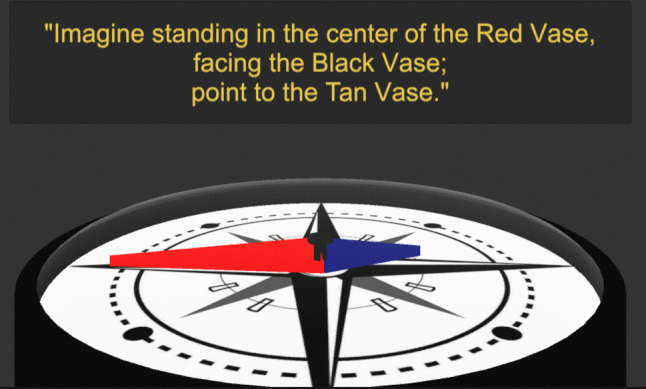
Example of the desktop JRD task

Within the JRD task, participants had to remember the directional spatial relations among objects in the environment. The task required participants to point in the direction of a target object from an imagined standing location and facing direction (“Imagine standing in the center of the X, facing the Y; point to the Z”). All participants experienced the same instruction order of 28 target triads that were chosen by the researcher from the larger 336 total possible triads. Triads were chosen to give participants a varied number of possible angles and room-based orientations (i.e., within rooms and between rooms). The pointing instructions remained visible for the duration of every trial, and below them was a visually rendered compass that began each trial in a 0-degree orientation (facing away/forward/North from the participant). To rotate the compass to match their desired pointing direction, participants had to press and hold either the left- or right-arrow key to rotate the longer red portion of the compass needle either counterclockwise or clockwise respectively (Fig. 5). Once they were satisfied with their response, they pressed the enter key to move to the next trial. At the beginning of the task, participants were given instructions on how the JRD operates on a general principle (i.e., standing at one location, facing a second, and then pointing to a third) and a real-world example similar to what they would experience in the trials (e.g., “imagine standing at the computer, facing the opposite wall, now point to the door”). As in the map-making task, participants were also informed that their time to complete each trial was not being recorded.

### Post experimental debriefing

Once participants had completed the experiment, they were first asked to freely respond to the following question: “What, if anything, did you notice about the space?” This question was asked to get a self-reported measure of whether any participants within the non-Euclidean condition noticed the impossible overlap as they navigated, drew their maps, or pointed from their memories. No participant said anything about the impossibility and instead made comments about what they saw within the virtual space (e.g., they would talk about the objects, paintings, or the textures, but not about the actual layout itself). After participants responded to the question, they were then debriefed with the paradigm fully explained.

### Data analysis

Data processing and analyses were completed in Excel 365 (Microsoft Corporation, Redmond, WA, United States), Matlab 2023b (MATLAB, 2023), RStudio (Posit Software, PBC, Boston, MA, United States), and the Gardony Map Drawing Analyzer (Gardony et al., 2016). To examine the trends in the raw data more in-depth, we looked at the timing and accuracy data for the learning and probe trials as well as the absolute pointing error from the JRD task. Specifically, we determined significant main effects using ANOVAs with parametric Welch’s t-tests to perform pairwise comparisons of our grouped pointing data across different pointing response types. Responses for each dependent variable that exceeded the upper or lower outer fences (Tukey, 1977) were defined as outliers and removed. A total of 2.93% of the training data, 6.13% of the recall data, and 2.29% of the pointing data were removed. No map data were removed. We used the Gardony Map Drawing Analyzer to compare the unique measures it provides based on pairwise comparisons between map landmarks, as well as bidimensional regression parameters (Gardony et al., 2016). Lastly, all Bayes Factors were calculated using the BayesFactor package for R (Morey & Rouder, 2024).

## Results

### Sex & testing order

We found no main effects on any measures based on grouping by sex (*F*(1,56) = 0.63, *p* = 0.43, BF_10_ = 0.14) or testing order (*F*(1,56) = 2.83, *p* = 0.10, BF_10_ = 0.17). We also found no interactions between any independent variables (room type x sex: *F*(1,56) = 1.34, *p* = 0.25, BF_10_ = 0.03; room type x testing order: *F*(1,56) = 0.02, *p* = 0.89, BF_10_ = 0.007; sex x testing order: *F*(1,56) = 0.21, *p* = 0.65, BF_10_ = 0.03; room type x sex x testing order: *F*(1,56) = 1.50, *p* = 0.23, BF_10_ = 0.005) Therefore, all analyses will focus on the differences between the Euclidean and Non-Euclidean conditions (room type: *F*(1,56) = 4.76, *p* = 0.04, BF_10_ = 0.21).

### Demographics

We found no significant differences based on performance measures, demographic information, and group (see Supplementary Materials).

### Training

**Fig. 6:**
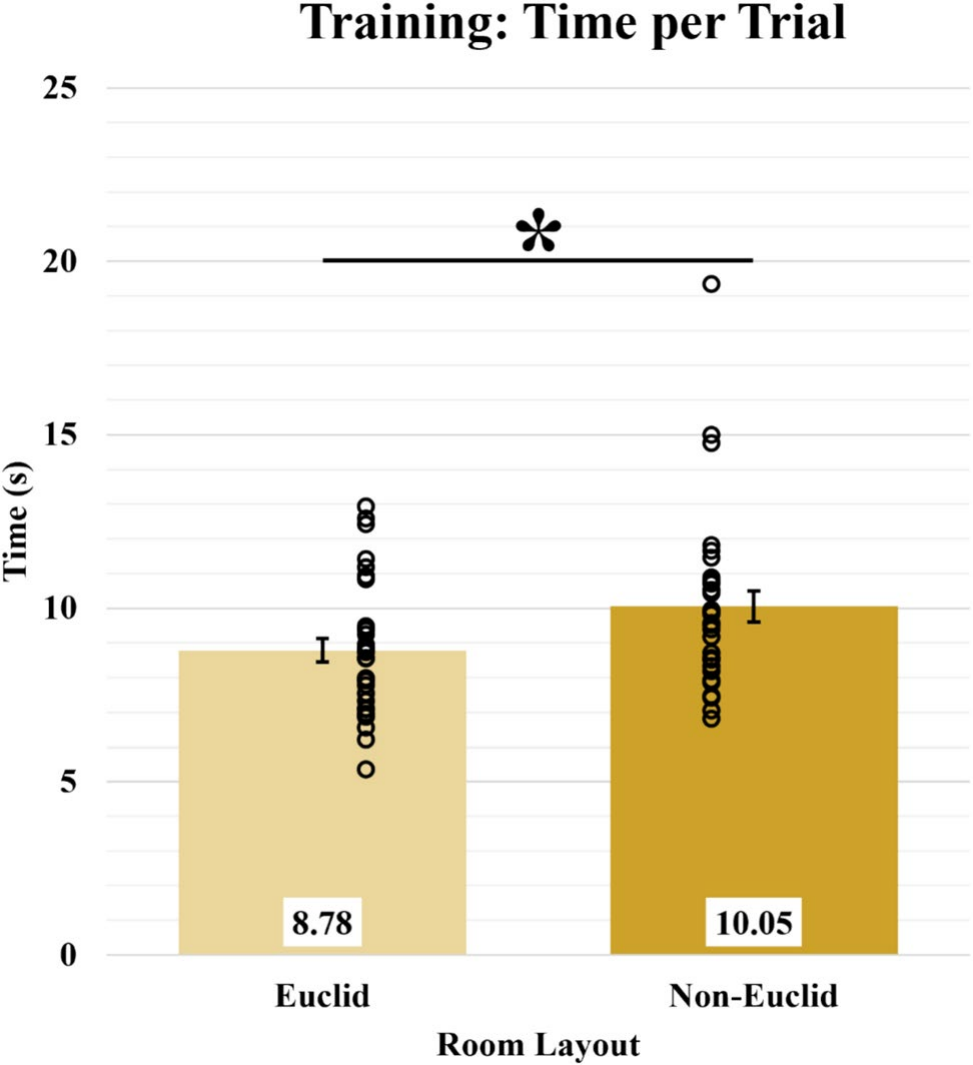
Average time in seconds per training trial for Euclidean and non-Euclidean participants, Open circles represent participants’ averaged data. Error bars represent SEM

To determine if the participants learned the environment with similar proficiency, we analyzed the average time per trial (Fig. 6). If the Euclidean condition took significantly longer than the non-Euclidean condition on the time required to complete the training task, a task that traditionally serves as a control for motivation/sensorimotor deficits due to its simplicity (see McAvan et al., 2021, 2022 for examples), then we would have to re-examine our paradigm. If the non-Euclidean condition took significantly longer than the Euclidean condition, then this early difference may provide an insight into which stage of spatial learning (e.g., encoding) is sensitive to interference by heuristic knowledge of Euclidean space. If both conditions took the same amount of time, then one could reason that they are encoding the spatial information at equal rates or with equal difficulty. An ANOVA demonstrated a significant difference in the average amount of time needed by the Euclidean condition (*M* = 8.78, *SD* = 1.92) and the non-Euclidean condition (*M* = 10.05, *SD* = 2.55) to complete the training task [*F*(1,62) = 5.05, *p* = 0.03, BF_10_ = 2.07].

### Recall

**Fig. 7:**
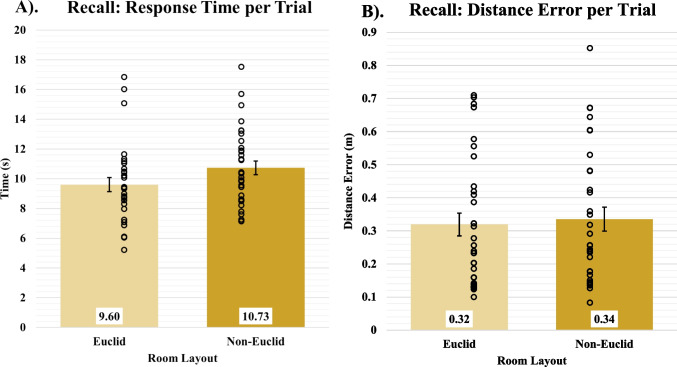
Recall data. **a**) Average time in seconds per recall trial for Euclidean and non-Euclidean participants. **b**) Average response distance (m) from the actual target location for Euclidean and non-Euclidean participants. Open circles represent participants’ averaged data. Error bars represent SEM

These analyses compared the conditions on their performance in the recall portion of the task. When comparing the temporal data, we found no significant difference between the Euclidean (*M* = 9.60, *SD* = 2.68) and non-Euclidean (*M* = 10.73, *SD* = 2.61) conditions [*F*(1,62) = 2.94, *p* = 0.09, BF_10_ = 0.87], demonstrating similar retrieval times for the invisible targets (Fig. 7a). When comparing the distance errors between the conditions, we again found no significant difference between the Euclidean (*M* = 0.32, *SD* = 0.19) participants and non-Euclidean (*M* = 0.34, *SD* = 0.20) conditions [*F*(1,62) = 0.11, *p* = 0.75, BF_10_ = 0.27] (Fig. 7b). These results show that while participants within the non-Euclidean condition took longer during training, once their knowledge of the space was encoded and ready for retrieval, they performed no differently from their control counterparts.

### Map making

To assess participants’ accuracy in their underlying cognitive spatial representations, we used the Gardony Map Drawing Analyzer (GMDA) to compare coordinate files generated by participants’ responses on the Map drawing task to the sets of coordinates from the layouts (i.e., Euclidean and non-Euclidean layouts). These comparisons allowed us to determine how well participant’s representations resembled their virtually experienced space. Using the GDMA tool, we calculated the following dependent measures for each participant:

Angle accuracy (*AA*) measures the accuracy of inter-object angles on the observed map. It is sensitive to the magnitude of angular error, not the sign. Absolute angular difference scores are calculated in degrees (0-180), summed, and divided by the number of object comparisons. This angular error score is then transformed to a proportion by dividing by 180 and subtracted from 1. Scores closer to 1 indicate more accurate inter-object angle representation.

The correlation coefficient, *r*, measures the extent of similarity between two configurations of points. It ranges from 0 to 1 (mathematically it is the magnitude of a vector, and hence, is never negative) and is insensitive to scaling, translation, and rotation of the observed map relative to the standard map. *r* ^2^ is the proportion of variance of the observed map’s configuration of locations accounted for by the standard map’s configuration of locations.

The distortion index (*DI*) denotes the percentage of distortion in the observed map and is reported as a percentage from 0 to 100 (Friedman & Kohler, 2003; Waterman & Gordon, 1984). *DI* ^2^ is the proportion of variance of the observed map’s location configuration that remains unexplained by bidimensional regression (BDR). *DI* relates to *r* ^2^ by the following equation: (*DI*/100)^2^ = 1- *r* ^2^ (Gardony, et al., 2016 pp. 158-161).

The procedures and formulae for calculating GMDA-unique measures (Angular Accuracy) can be found in Appendix A of Gardony, et al., 2016 and the BDR equations (R^2^ and DI) can be found in Friedman & Kohler, 2003.

**Fig. 8:**
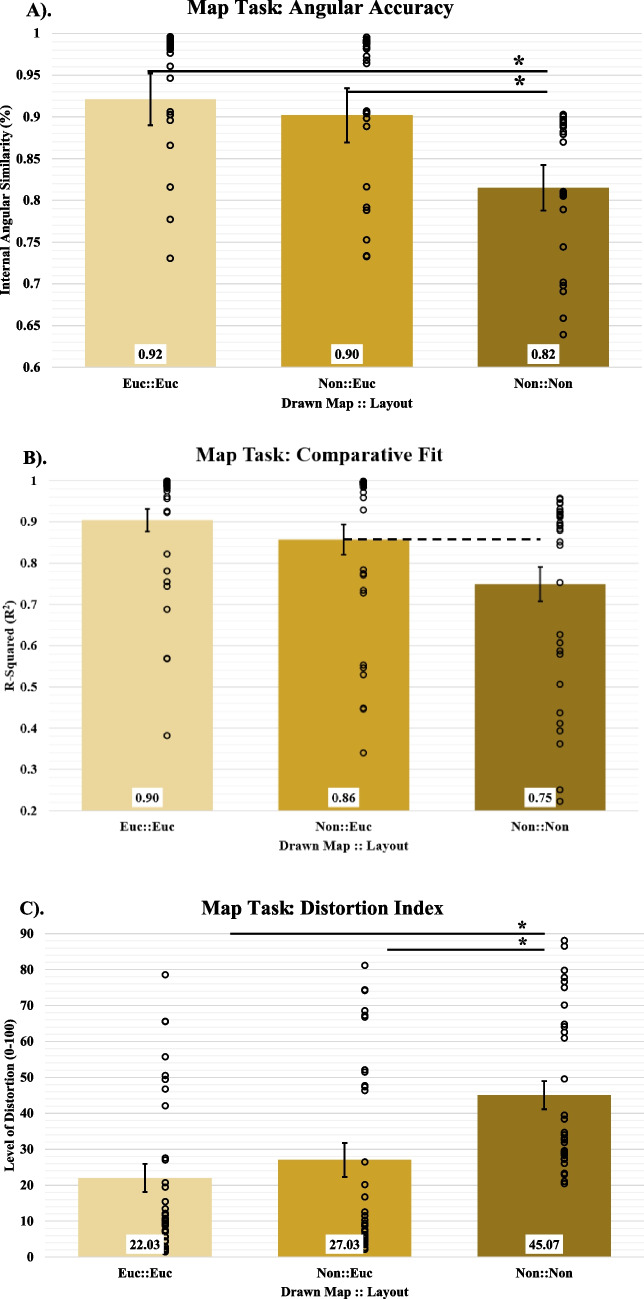
Map Task Parameters When Comparing Euclidean Maps to the Euclidean Layout, Non-Euclidean Maps to the Euclidean Layout, and Non-Euclidean Maps to the Non-Euclidean Layout. **a**) Average Angular Accuracy score across map comparisons. **b**). Average R-squared across map comparisons. **c**). Average Distortion Index across map comparisons. Open circles represent participants’ average data. Error bars represent SEM

Comparing the Euclidean condition’s maps to the actual Euclidean layout yielded a good fit on all dependent measures (AA_Mean_ = 0.92, AA_SD_ = 0.18, R^2^_Mean_ = 0.90, R^2^_SD_ = 0.16, DI_Mean_ = 22.02, DI_SD_ = 22.12). Comparing the non-Euclidean condition’s maps to the actual non-Euclidean layout yielded poor fit (AA_Mean_ = 0.82, AA_SD_ = 0.16, R^2^_Mean_ = 0.75, R^2^_SD_ = 0.23, DI_Mean_ = 45.07, DI_SD_ = 22.17); the comparison of the non-Euclidean condition’s maps to the actual Euclidean layout yielded a better fit (AA_Mean_ = 0.90, AA_SD_ = 0.18, R^2^_Mean_ = 0.86, R^2^_SD_ = 0.21, DI_Mean_ = 27.03, DI_SD_ = 26.88). The non-Euclidean condition’s maps fit the Euclidean layout significantly better than the non-Euclidean layout for angular accuracy and distortion index [*t*_*AA*_(60) = 2.04, *p*_*AA*_ < 0.05, BF_10_ = 1.46; *t*_*R2*_(61) = 1.95, *p*_*R2*_ = .056, BF_10_ = 1.25; *t*_*DI*_(60) = 2.93, *p*_*DI*_ < .001, BF_10_ = 8.45]. These results show that participants in the non-Euclidean condition tended to make maps that were significantly more like Euclidean space than non-Euclidean space (see Fig. 8).

As further evidence for this conclusion, we also found no significant differences between the Euclidean condition’s fits and the non-Euclidean condition’s fits when both were compared to the Euclidean layout [*t*_*AA*_(62) = 0.43, *p*_*AA*_ = 0.67, BF_10_ = 0.28; *t*_*R2*_(58) = 1.03, *p*_*R2*_ = 0.31, BF_10_ = 0.40; *t*_*DI*_(58) = 0.89, *p*_*DI*_ = 0.37, BF_10_ = 0.34]. Overall, these results suggest that when all participants recreated maps of the environment, they did so in a way that conformed more with Euclidean norms and expectations than not. And, from a qualitative standpoint, no participant demonstrated the inversion of the central objects in the non-Euclidean virtual environment, leading us to conclude that when encountering these non-Euclidean spaces, participants were unable to reconcile the conflicting information and instead fell back on a heuristic representation of space that conformed to Euclidean geometric properties.

To further assess possible distortions in participants’ mental representations of the layouts, we compared the ratios of distances along the x and the y axes (where x axis is parallel to the long side of the rectangular spaces shown in Fig. 1) for the room layouts and the reconstructed maps. In the Euclidean layout (Fig. 1a), the aspect ratio of the quadrilaterals formed by the four objects in each sub-room is .95 (x/y). In the non-Euclidean layout (Figs. 1b & c), the corresponding aspect ratio, computed for each sub-room, is 1.31. To determine these rectangular measurements in reconstructed maps, we measured the distances between the four pairs of objects that formed each of the four sides of the appropriate quadrilaterals and then averaged those values across parallel sides and across sub-rooms, yielding one distance along each of the x and y axes for each participant. The reconstructed aspect ratio was computed from these values.

**Fig. 9:**
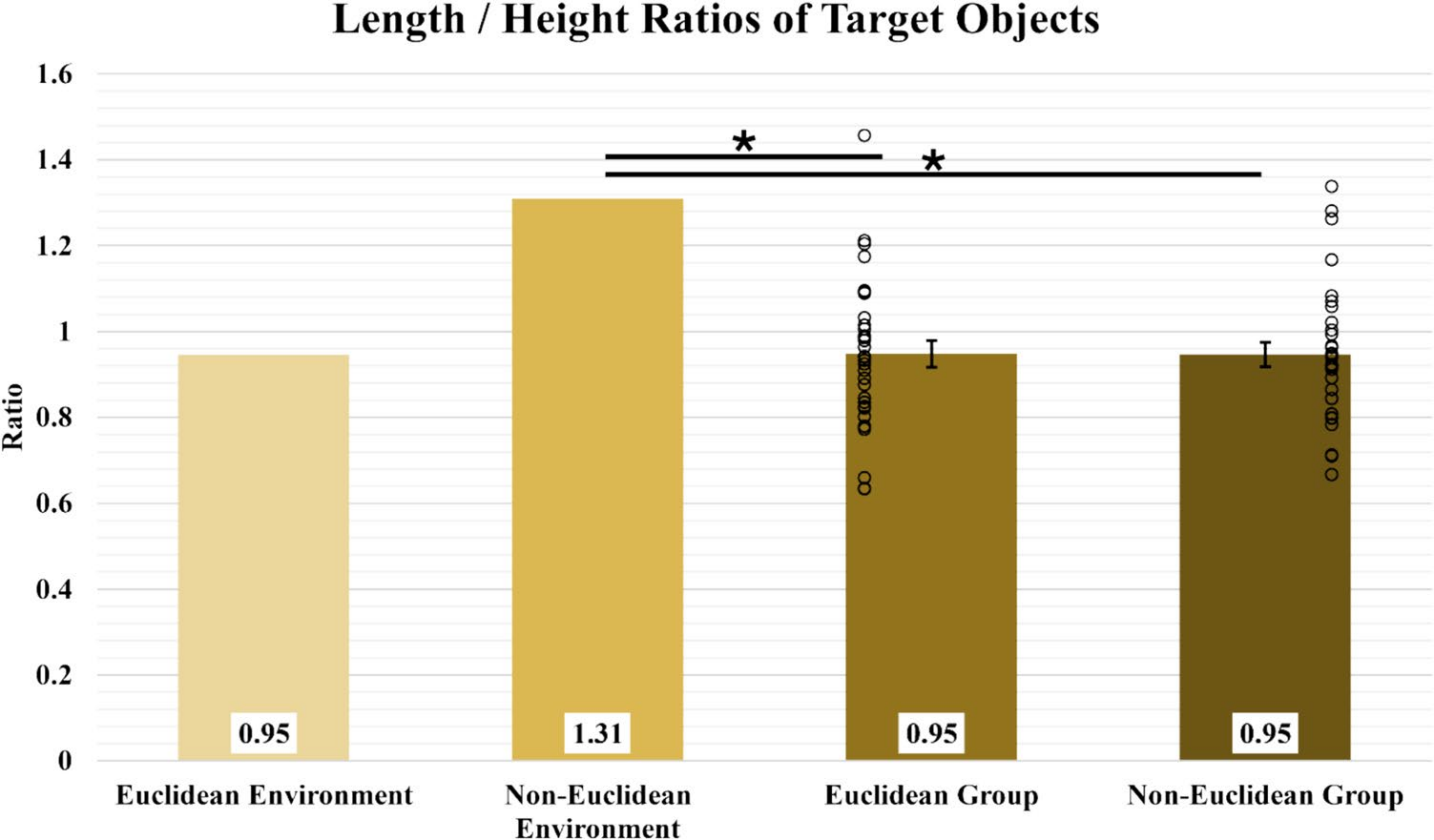
Length/height ratios (rectangular measurements) of target objects across environments and conditions. Open circles represent participants’ average data. Error bars represent SEM

There were no significant differences between the ratios of the Euclidean and Non-Euclidean conditions (E_Mean_ = 0.95, E_SD_ = 0.03, N_Mean_ = 0.95, N_SD_ = 0.03, *t*(61) = 0.03, *p* = 0.98, BF_10_ = 0.26). Furthermore, neither condition differed significantly from the Euclidean environment’s ratio of 0.95 (*t*_EE_(31) = 0.07, *p*_EE_ = 0.94, BF_10_ = 0.26; *t*_NE_(31) = 0.04, *p*_NE_ = 0.97, BF_10_ = 0.26), but both differed significantly from the non-Euclidean environment’s ratio of 1.31 (*t*_EN_(31) = 11.65, *p*_EN_ < 0.001, BF_10_ = 1.19e14; *t*_NN_(31) = 12.89 *p*_NN_ < 0.001, BF_10_ = 8.88e15). These results show that participants in the non-Euclidean condition did **not** preserve the aspect ratio of the non-Euclidean layout they experienced and instead mentally represented the two sub-rooms as nearly square (Fig. 9). We examine possible causes of this in the Discussion section.

#### JRD

**Fig. 10:**
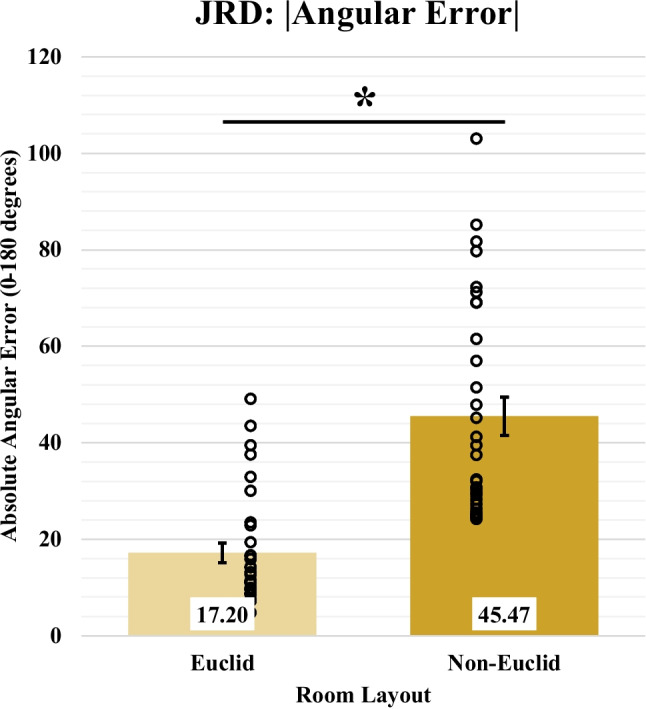
Absolute Pointing Error compared between Euclidean and non-Euclidean conditions. Open circles represent participants’ averaged data. Error bars represent SEM

For the JRD task, absolute pointing error was calculated as the absolute value of the difference between the correct response given by the object triads and the response made by the participants. When comparing the average absolute pointing error between the Euclidean condition (*M* = 17.20, *SD* = 11.71) and non-Euclidean condition (*M* = 45.47, *SD* = 22.50), we found that the Euclidean condition had a significantly lower average absolute pointing error [*F*(1,62) = 39.75, *p* < 0.001, BF_10_ = 3.00e5]. This result shows that non-Euclidean participants perform significantly worse on the JRD than their Euclidean counterparts (Fig. 10).

**Fig. 11:**
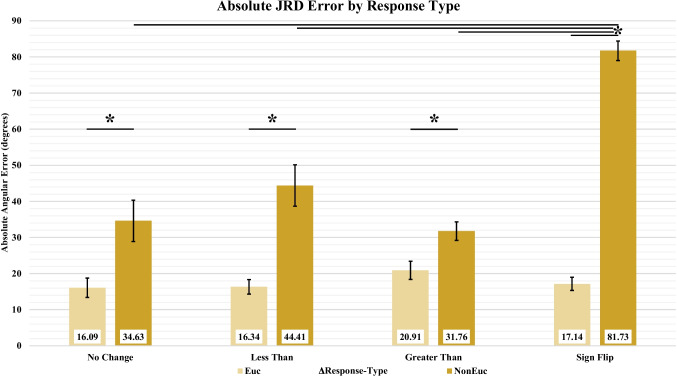
Absolute pointing error by change in response type. Error bars represent SEM

We classified the JRD responses into response types based on how the correct angle would change between the Euclidean and the non-Euclidean layouts. This generated four separate types: no change responses, less-than responses (smaller in non-Euclidean environment), greater-than responses (larger in non-Euclidean environment), and sign-flip responses (180-degree difference between the rooms). We then separated and averaged participants’ responses per each response type and compared the absolute angular error across types (Fig. 11). Responses from the Euclidean condition did not differ significantly across response types (*t*_*no/less*_*(*57) = 0.07, *p*_*no/less*_ = 0.94, BF_10_ = 0.26; *t*_*no/great*_*(*62) = 1.29, *p*_*no/great*_ = 0.20, BF_10_ = 0.28; *t*_*no/flip*_(55) = 0.32, *p*_*no/flip*_ = 0.75, BF_10_ = 0.28; *t*_*less/great*_*(*59) = 1.39, *p*_*less/great*_ = 0.17, BF_10_ = 0.27; *t*_*less/flip*_*(*62) = 0.29, *p*_*less/flip*_ = 0.77, BF_10_ = 0.30; *t*_*great/flip*_(57) = 1.18, *p*_*great/flip*_ = 0.24, BF_10_ = 0.41). In the non-Euclidean condition, pointing error was significantly higher for the sign-flipped type than for the other response types (See Table 1; *t*_*no/flip*_*(*44) = 7.32, *p*_*no/flip*_ < 0.001, BF_10_ = 1.38e6; *t*_*less/flip*_*(*44) = 5.81, *p*_*less/flip*_ < 0.001, BF_10_ = 5.03e4; *t*_*greater/flip*_*(*62) = 13.14, *p*_*greater/flip*_ < 0.001, BF_10_ = 2.06e16). There were no significant differences across the other response types within the non-Euclidean condition (*t*_*no/less*_*(*62) = 1.19, *p*_*no/less*_ = 0.24, BF_10_ = 0.35; *t*_*no/great*_*(*43) = 0.45, *p*_*no/great*_ = 0.65, BF_10_ = 0.34; *t*_*less/great*_* (*43) = 1.99, *p*_*less/great*_ = 0.053, BF_10_ = 1.33). Finally, pointing error was significantly greater in the non-Euclidean condition than in the Euclidean condition for all response types (*t*_*no/no*_*(*44) = 2.89, *p*_*no/no*_ < 0.01, BF_10_ = 1.52; *t*_*less/less*_*(*38) = 4.57, *p*_*less/less*_ < 0.001, BF_10_ = 17.07; *t*_*great/great*_*(*62) = 3.07, *p*_*great/great*_ < 0.005, BF_10_ = 0.95; *t*_*flip/flip*_*(*55) = 19.33, *p*_*flip/flip*_ < 0.001, BF_10_ = 2.01e19). All statistically significant comparisons survived Bonferroni multiple-comparison corrections.

**Table 1:** Absolute pointing error per condition by response change type

	No Change		Less Than		Greater Than		Sign Flip	
Euclidean	M = 16.09	SD = 15.11	M = 16.34	SD = 11.28	M = 20.91	SD = 14.38	M = 17.14	SD = 10.44
Non-Euclidean	M = 34.63	SD = 32.37	M = 44.41	SD = 32.31	M = 31.76	SD = 14.55	M = 81.73	SD = 15.39

### Post-hoc correlates of performance

**Fig. 12:**
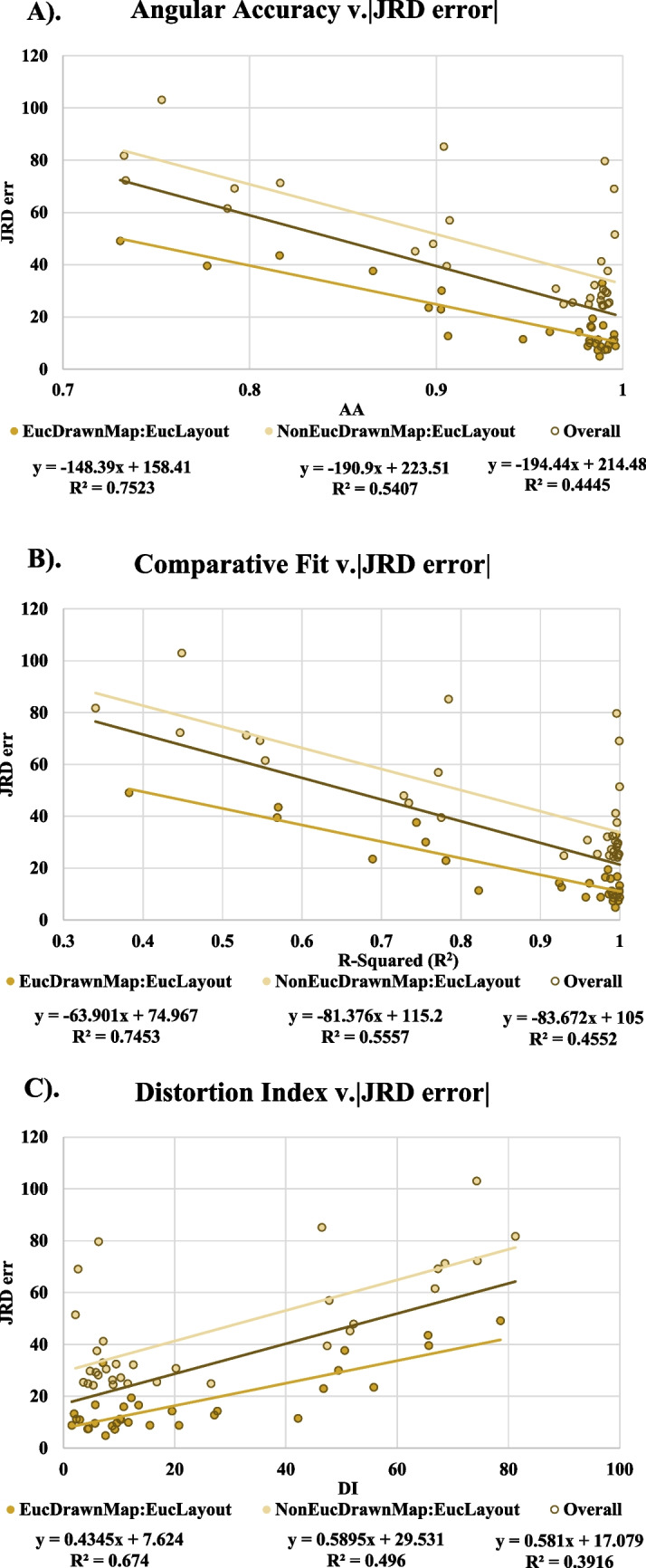
Absolute JRD Pointing Error correlated with map-drawing measures between and across conditions. a). Absolute JRD Pointing Error correlated with Angular Accuracy. b). Absolute Pointing Error correlated with map R^2^. c) Absolute JRD Pointing error correlated with map distortion index. Dots represent participant responses

We correlated all dependent measures (training time, recall time, recall distance error, JRD absolute angular error, map-drawing AA, R2, & DI) and found significant correlations between all Euclidean-comparison map-drawing measures and JRD performance (Fig. 12), showing that those with better drawn maps performed better on the JRD pointing task and vice versa. This supports the idea that both measures are examining the same underlying spatial representation (Huffman & Ekstrom, 2019).

## Discussion

We found that, when presented with a non-Euclidean virtual space that is impossible to experience in the real world, participants did not seem to form a flexible cognitive graph representation of the space but instead reverted to their Euclidean representations of space, forming an *incorrect* cognitive map. Their drawn maps almost mirrored the Euclidean layout and differed significantly from the experienced non-Euclidean layout. The analysis of the aspect ratios of the quadrilaterals defined by the four objects in each sub-room yielded the same pattern of results. Pointing errors in the JRD task were higher overall in the non-Euclidean condition than in the Euclidean condition and were especially high for object triads that had nearly opposite directional relations in the non-Euclidean and the Euclidean environments.

Taken together, these findings show that participants in the non-Euclidean condition mentally represented the two sub-rooms to be consistent with the enclosing environment, the extent of which they experienced visually and by walking from the door of one room to the door of the other. Put another way, their experiences in the rectangular, overlapping sub-rooms were represented as nearly-square, non-overlapping sub-rooms within the larger environment.

It would have been possible for participants in the non-Euclidean condition to represent the two sub-rooms as those rooms were experienced (i.e., rectangular) and as non-overlapping. But such a representation of two non-overlapping rectangular rooms would be inconsistent with the dimensions of the sub-rooms relative to the larger space (i.e., the total volume of both sub-rooms combined is greater than the volume of the space which contains them). It is intriguing that the experienced size of the larger environment affected how the smaller sub-rooms were mentally represented. The larger room might have been privileged because its extent was experienced before the extents of the sub-rooms (participants started in the hallway). Participants also walked farther when traversing the hallway between sub-rooms than when locomoting within each sub-room. This may indirectly point to the important role of self-motion and path integration in forming mental representations of the environment (Wang, 2016).

This behavior of mis-representing the true non-Euclidean space in favor of a false heuristic-based Euclidean space is broadly consistent with previous studies of spatial memory. Tversky (1981) may provide one of the first examples of people forming perceptual groups in spatial memory based on preconceived notions. She found that even when participants were explicitly forewarned about heuristic-based errors, they still formed perceptual groups based on alignment and proximity. McNamara (1986) also found that participants formed cognitive groups of spatial objects based on which delineated sub-region of the constructed environment they were located. Widdowson and Wang (2022) found that when pointing on hyperbolic and spherical surfaces, participants would fail to account for the curvature of the space and instead treated the readily apparent non-Euclidean space as if it were Euclidean, demonstrating a systematic bias to Euclidean norms even with perceptual experience. Kim and Doeller’s (2024) results further exemplify this effect by showing that regardless of whether participants navigated in a Euclidean or non-Euclidean space, their spatial representations were more closely aligned with utilizing multiple two-dimensional flat-plane maps (Euclidean) as opposed to a single three-dimensional volumetric representation (non-Euclidean) even when the former would be less accurate for the actual space than the latter.

We found that training times in the Euclidean and non-Euclidean conditions differed significantly, whereas recall times and distance errors were similar. This difference in training performance leads us to posit that the reliance on a Euclidean heuristic occurs early in spatial learning and likely impacts the encoding phase. Although post-experimental debriefings indicated that participants in the non-Euclidean condition were not consciously aware of the spatial inconsistencies that they had experienced, the difference in training times indicates that extra cognitive processing might have been occurring. We speculate that participants were using a Euclidean prior schema to interpret their spatial experiences and that this process required additional cognitive effort. The resulting mental representation was internally consistent and permitted comparatively efficient recall of the locations of objects within each sub-environment, while increasing errors when recalling the locations of objects between the two sub-environments.

Participants’ absence of awareness of non-Euclidean manipulations, unless they were explicitly informed by researchers or the manipulation exceeded 50% of the virtual space, has also been observed previously (Ciumedean et al., 2020; Suma, et al., 2012; Vasylevska & Kaufmann, 2015). Because non-Euclidean violations are essentially impossible to experience in everyday life, people might not have the cognitive processes to adequately recognize and form a mental representation of non-Euclidean space, instead reverting to their a priori knowledge of Euclidean space. In other words, people may not be able to reconcile impossible information in their minds and as such default to what *should* be possible.

Although fully-immersive VR is not as visually rich as the real-world and people may mis-represent aspects of virtual environments (Kelly et al., 2017a, 2017b; Kelly et al., 2018; Zhao et al., 2023), our results lead us to conclude that when presented with an impossible, non-Euclidean space, naïve participants are unable to flexibly encode the space in the form of a cognitive graph and instead alter their representations to closer match their Euclidean expectations and form an inaccurate cognitive map. Specifically, people maintain locally cohesive Euclidean sub-maps on a globally inconsistent primary map.

### Limitations

Our study showed that a 1 m central overlap was not noticed by participants in the non-Euclidean condition, yet it affected the encoding of the spaces (as indicated by training times) and their mental representations (as revealed in map making and JRD). It would be informative to parametrically vary the extent of overlap of the two sub-rooms to determine the psychophysical threshold at which the overlap is noticed. Preliminary work in our lab indicates that an overlap as large as 50% remains unnoticed.

Another limitation is that we did not assess or manipulate participants’ prior experience with non-Euclidean, impossible spaces. It is possible that people with extensive experience with and knowledge of these spaces would perform significantly better than those without.

It is also important to note that while we hypothesize that regression to a Euclidean heuristic occurs early in spatial learning and likely impacts the encoding phase, this conjecture is not directly tested in the current project.

## Supplementary Information

Below is the link to the electronic supplementary material.

Supplementary file1 (ZIP 85420 KB)

## Data Availability

Data transparency: All data and materials are available upon request.
