# Supplementary material for: Mental Representations of Impossible Non-Euclidean Environments
Source: Psychol Res. 2026 May 13;90(3):91. doi: 10.1007/s00426-026-02297-3 (PMC13171680; doi:10.1007/s00426-026-02297-3)
Supplement: Supplementary file 1 — Supplementary file1 (ZIP 85420 KB) [file 426_2026_2297_MOESM1_ESM.zip › SupplementalMaterials/SpatRepsONEEs_SupplementalAnalyses.docx]

**Supplemental Materials**

**Demographics**

|  | *E_Age* | *N_Age* |  | *E_SBSOD* | *N_SBSOD* |  | *E_VRSQ* | *N_VRSQ* |
| --- | --- | --- | --- | --- | --- | --- | --- | --- |
| Mean | 21.44 | 22.34 | Mean | 4.03 | 4.09 | Mean | 11.45 | 14.14 |
| Variance | 16.45 | 12.30 | Variance | 1.27 | 1.27 | Variance | 191.26 | 441.95 |
| Observations | 32 | 32 | Observations | 32 | 32 | Observations | 32 | 32 |
| Hypothesized Mean Difference | 0 |  | Hypothesized Mean Difference | 0 |  | Hypothesized Mean Difference | 0 |  |
| df | 61 |  | df | 62 |  | df | 54 |  |
| t Stat | -0.96 |  | t Stat | -0.24 |  | t Stat | -0.60 |  |
| P(T<=t) one-tail | 0.17 |  | P(T<=t) one-tail | 0.41 |  | P(T<=t) one-tail | 0.27 |  |
| t Critical one-tail | 1.67 |  | t Critical one-tail | 1.67 |  | t Critical one-tail | 1.67 |  |
| P(T<=t) two-tail | 0.34 |  | P(T<=t) two-tail | 0.81 |  | P(T<=t) two-tail | 0.55 |  |
| t Critical two-tail | 2.00 |  | t Critical two-tail | 2.00 |  | t Critical two-tail | 2.00 |  |
|  |  |  |  | *E_VGMH* | *N_VGMH* |  | *E_TE* | *N_TE* |
|  |  |  | Mean | 15.03 | 8.13 | Mean | 23.19 | 22.63 |
|  |  |  | Variance | 483.77 | 205.40 | Variance | 36.35 | 48.18 |
|  |  |  | Observations | 32 | 32 | Observations | 32 | 32 |
|  |  |  | Hypothesized Mean Difference | 0 |  | Hypothesized Mean Difference | 0 |  |
|  |  |  | df | 53 |  | df | 61 |  |
|  |  |  | t Stat | 1.49 |  | t Stat | 0.35 |  |
|  |  |  | P(T<=t) one-tail | 0.07 |  | P(T<=t) one-tail | 0.37 |  |
|  |  |  | t Critical one-tail | 1.67 |  | t Critical one-tail | 1.67 |  |
|  |  |  | P(T<=t) two-tail | 0.14 |  | P(T<=t) two-tail | 0.73 |  |
|  |  |  | t Critical two-tail | 2.01 |  | t Critical two-tail | 2.00 |  |

Table S1: t-tests for differences in demographics based on experimental group.

For all graphs, the Euclidean group is shown in blue and the non-Euclidean group in orange. Dots represent individual participants. Linear correlations are shown with the Euclidean group being on the left and the non-Euclidean group on the right.

**Santa Barbara Sense of Direction**

**Virtual Reality Sickness Questionnaire**

**Videogame Monthly Hours Played**

**Technology Exposure**
