# Supplementary figures and images for: Mental Representations of Impossible Non-Euclidean Environments
Source: Psychol Res. 2026 May 13;90(3):91. doi: 10.1007/s00426-026-02297-3 (PMC13171680; doi:10.1007/s00426-026-02297-3)

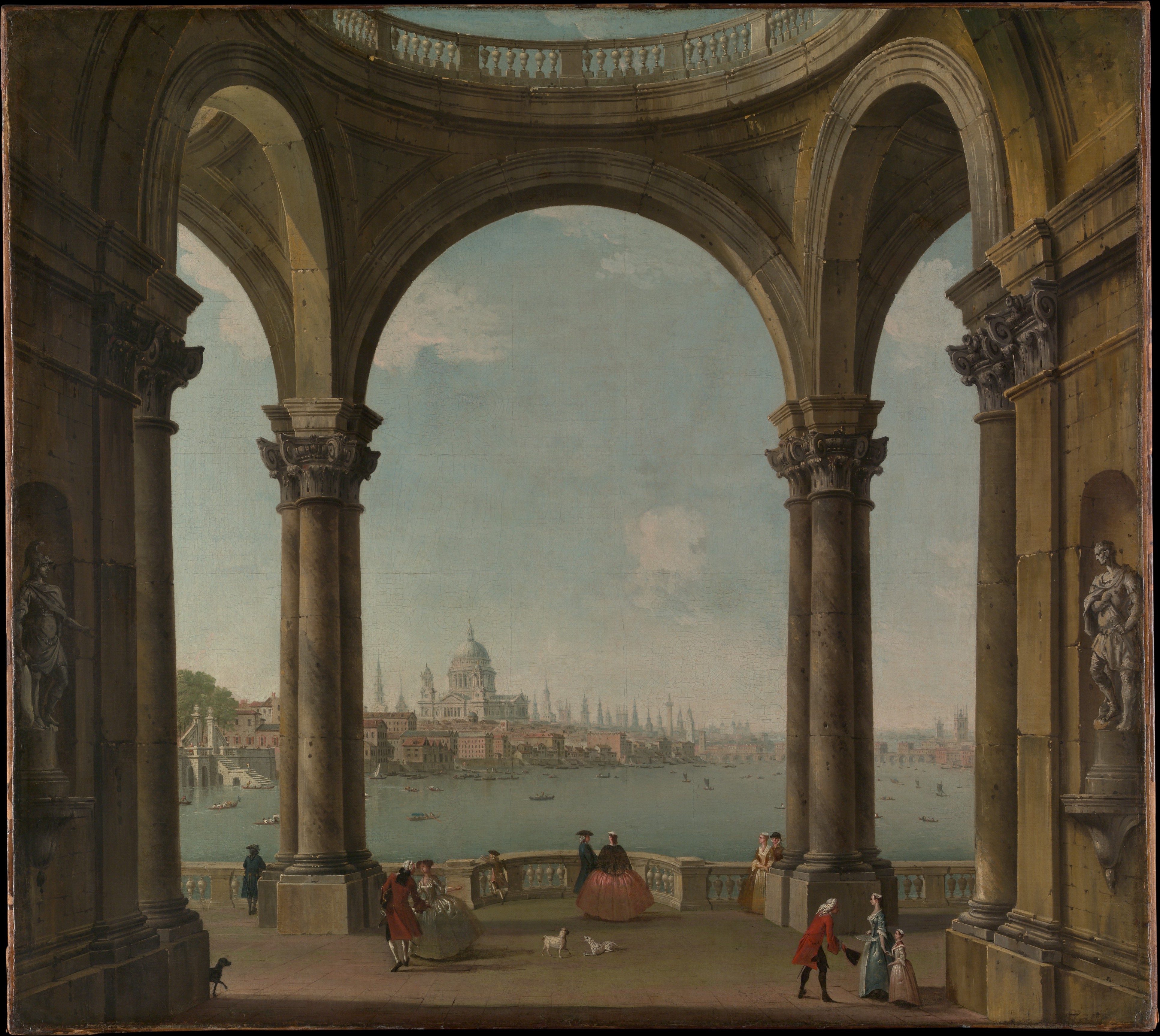

Supplement: Supplementary file 1 — Supplementary file1 (ZIP 85420 KB) [file 426_2026_2297_MOESM1_ESM.zip › SupplementalMaterials/NYMEt_OpenAccess_Textures/Cappriccio_with_St_Pauls_and_Old_London_Bridge_by_Antonio_Joli_106.7x119.4cm.jpg]

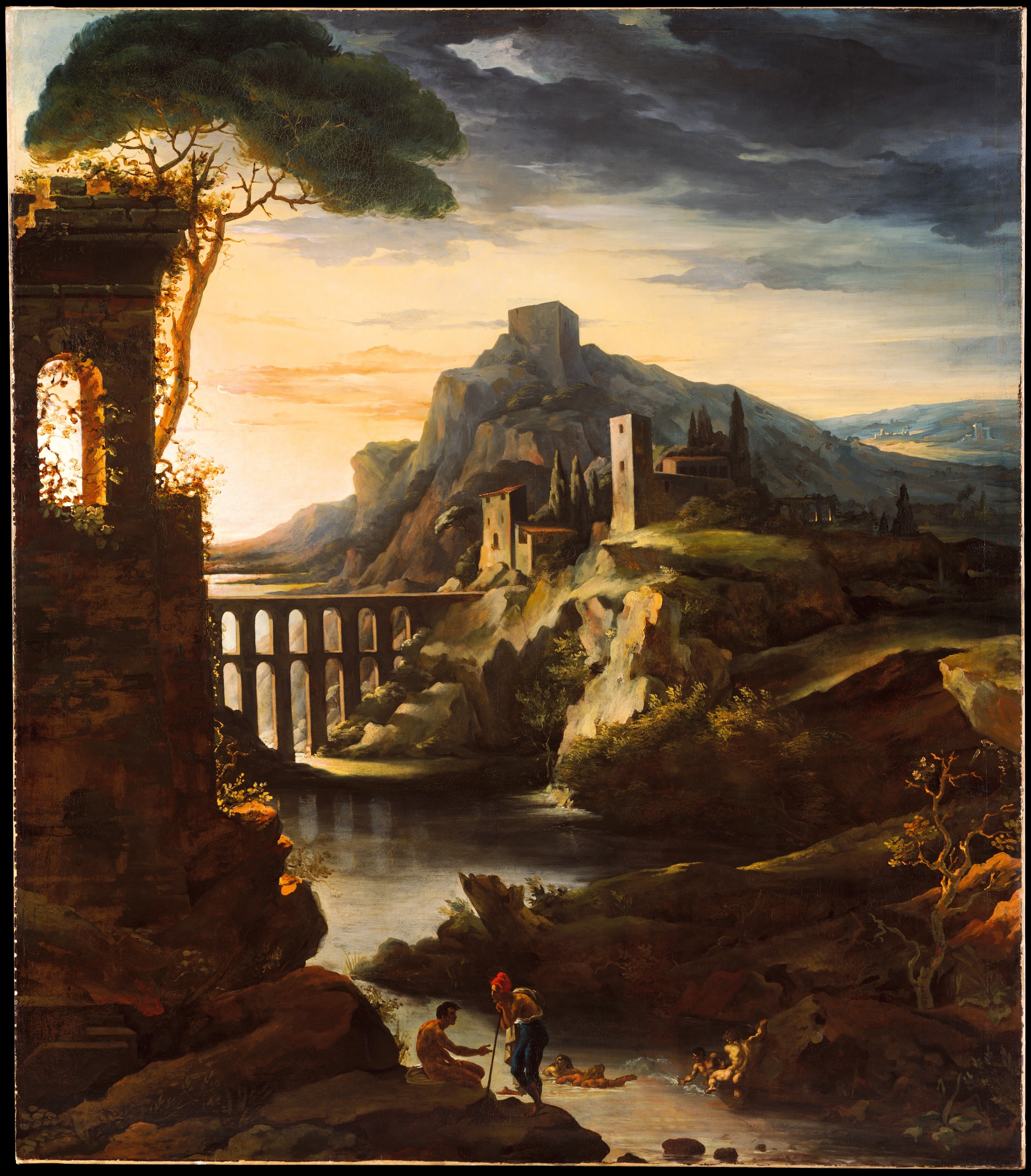

Supplement: Supplementary file 1 — Supplementary file1 (ZIP 85420 KB) [file 426_2026_2297_MOESM1_ESM.zip › SupplementalMaterials/NYMEt_OpenAccess_Textures/Evening_Landscape_with_an_Aqueduct_by_Theodore_Gericault_250.2x219.7cm.jpg]

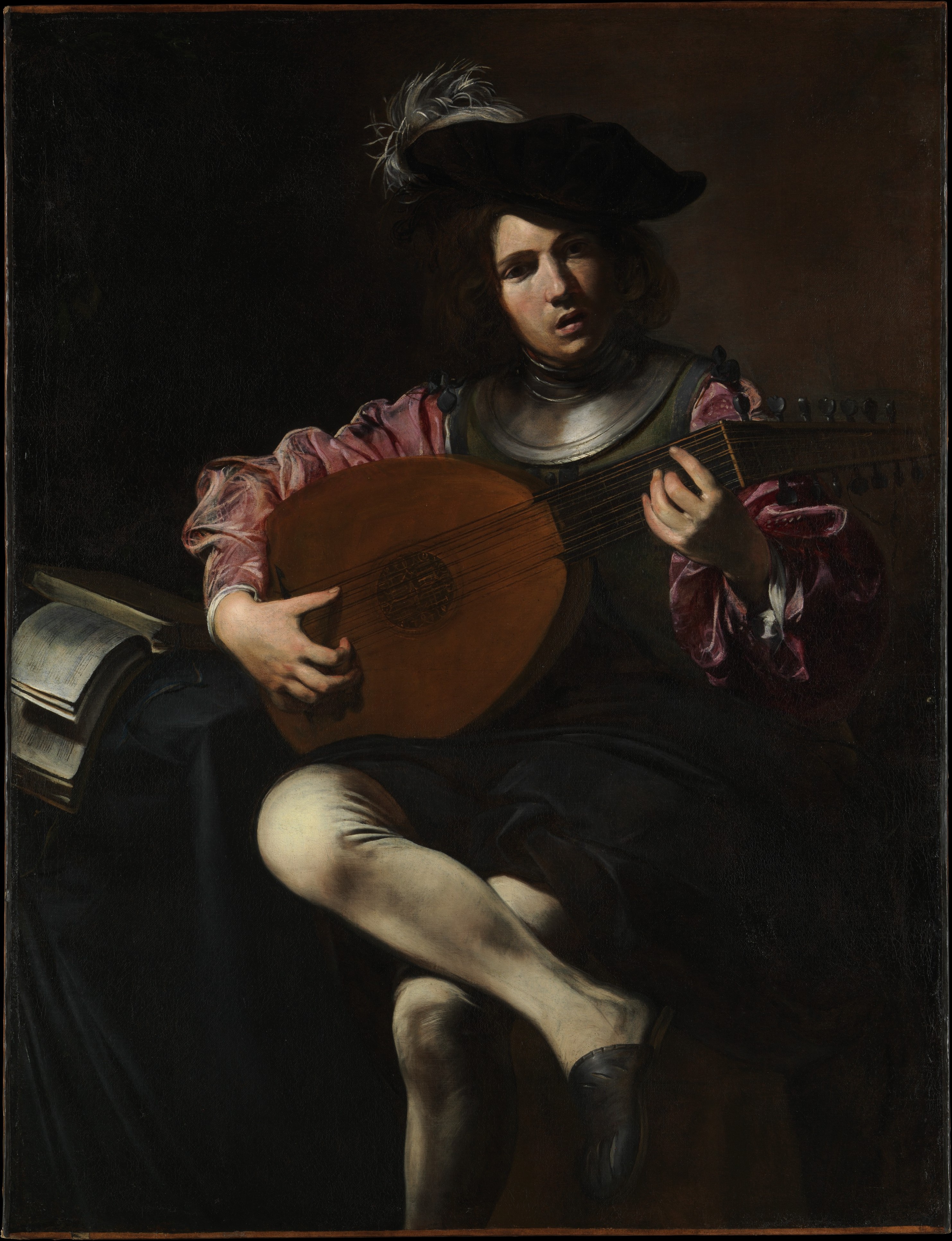

Supplement: Supplementary file 1 — Supplementary file1 (ZIP 85420 KB) [file 426_2026_2297_MOESM1_ESM.zip › SupplementalMaterials/NYMEt_OpenAccess_Textures/Lute_Player_by_Valentin_de_Boulogne_128.3x99.1cm.jpg]

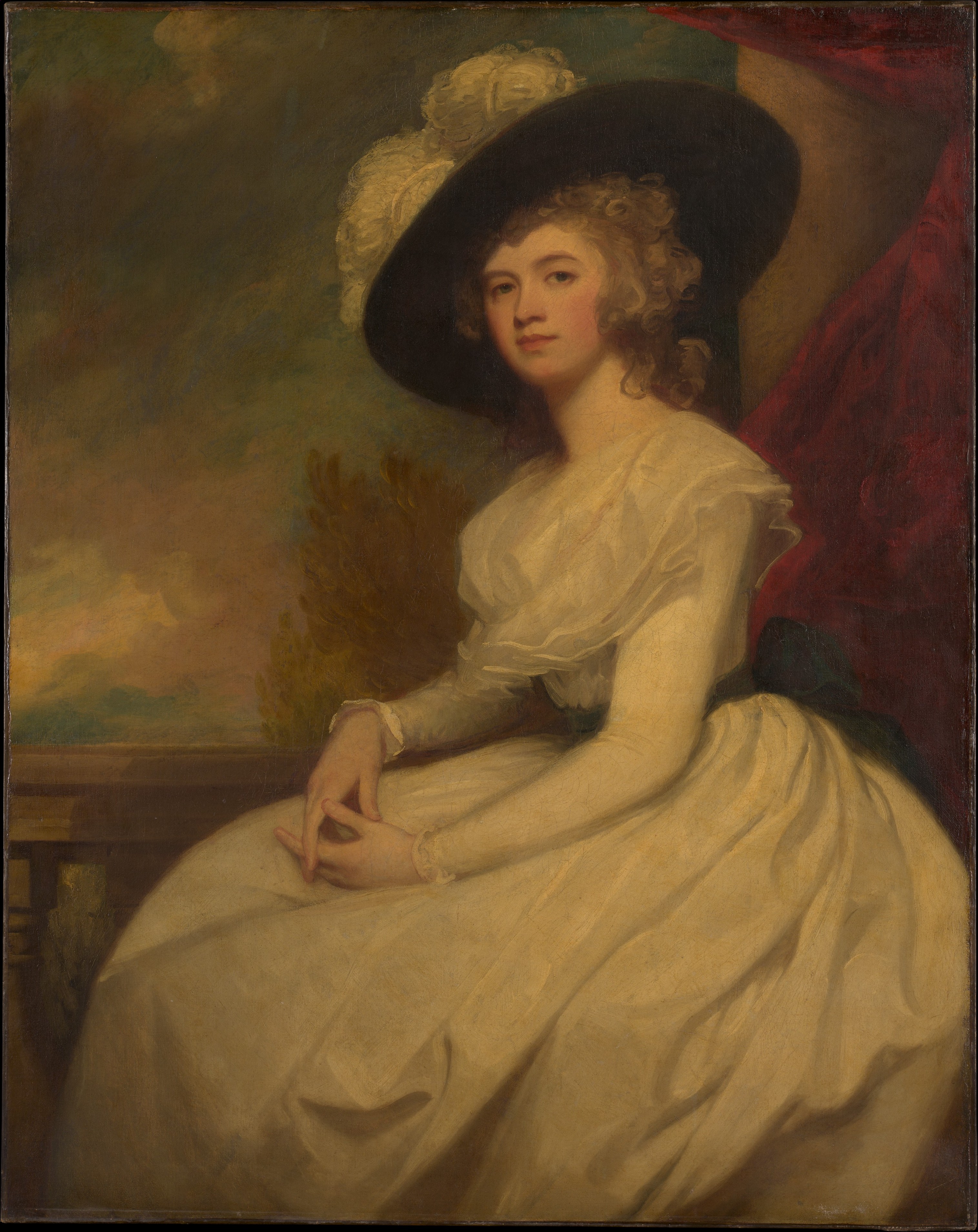

Supplement: Supplementary file 1 — Supplementary file1 (ZIP 85420 KB) [file 426_2026_2297_MOESM1_ESM.zip › SupplementalMaterials/NYMEt_OpenAccess_Textures/Mrs_Bryan_Cook_by_George_Romney_127x100.3cm.jpg]

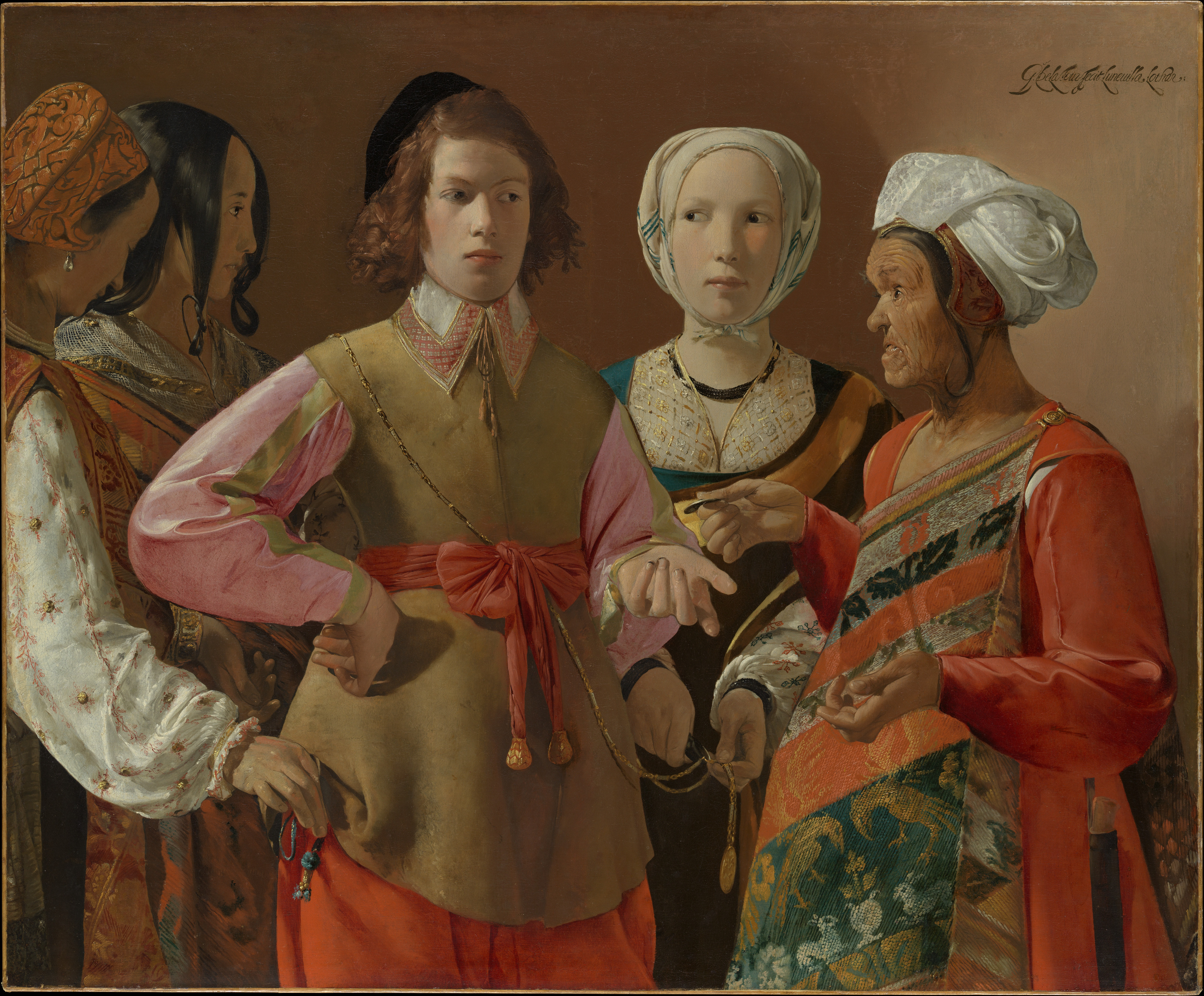

Supplement: Supplementary file 1 — Supplementary file1 (ZIP 85420 KB) [file 426_2026_2297_MOESM1_ESM.zip › SupplementalMaterials/NYMEt_OpenAccess_Textures/The_Fortune_Teller_by_Georges_de_La_Tour_101.9x123.5cm.jpg]

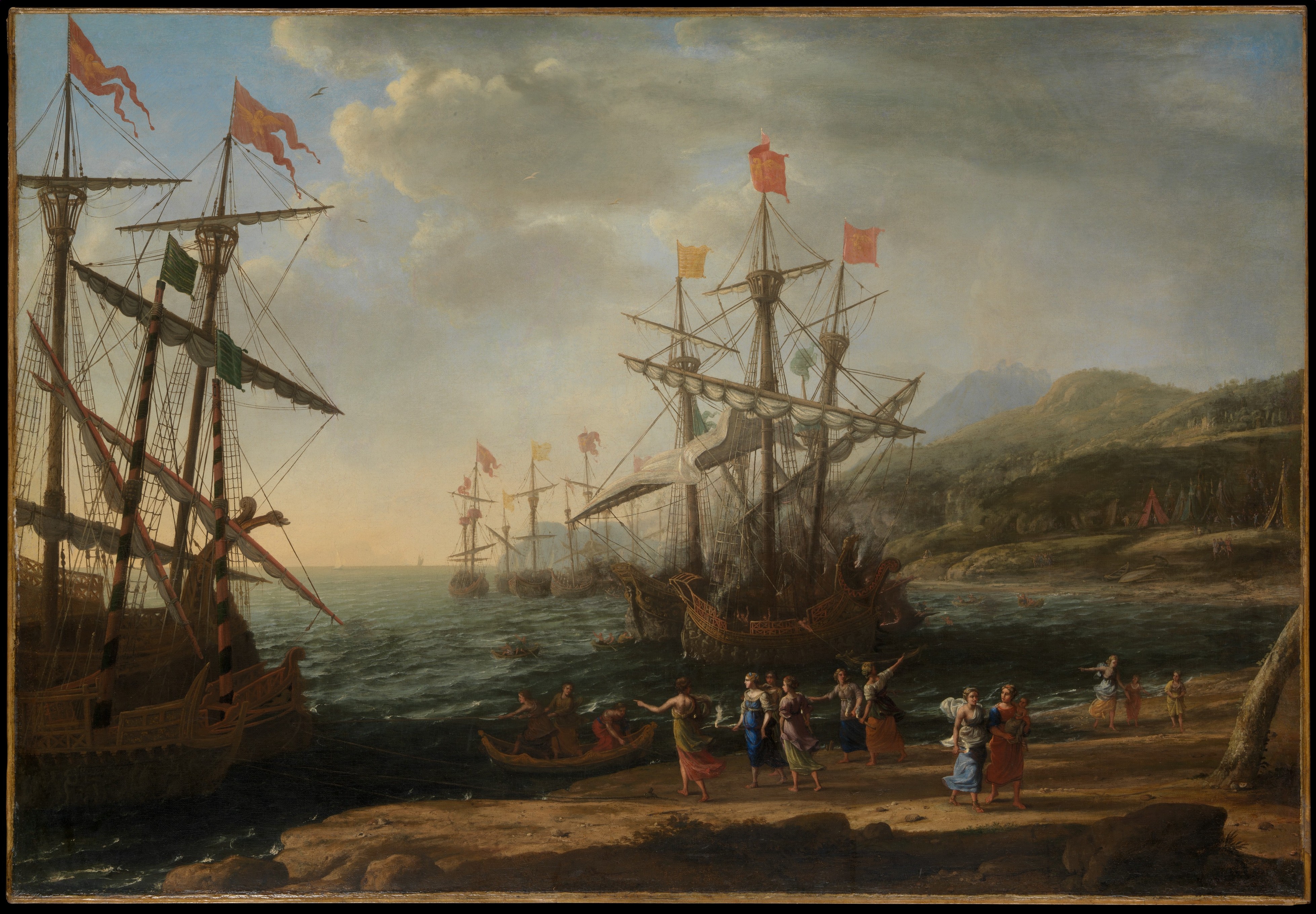

Supplement: Supplementary file 1 — Supplementary file1 (ZIP 85420 KB) [file 426_2026_2297_MOESM1_ESM.zip › SupplementalMaterials/NYMEt_OpenAccess_Textures/The_Trojan_Women_Setting_Fire_to_their_Fleet_by_Claude_Lorrain_105.1x152.1cm.jpg]

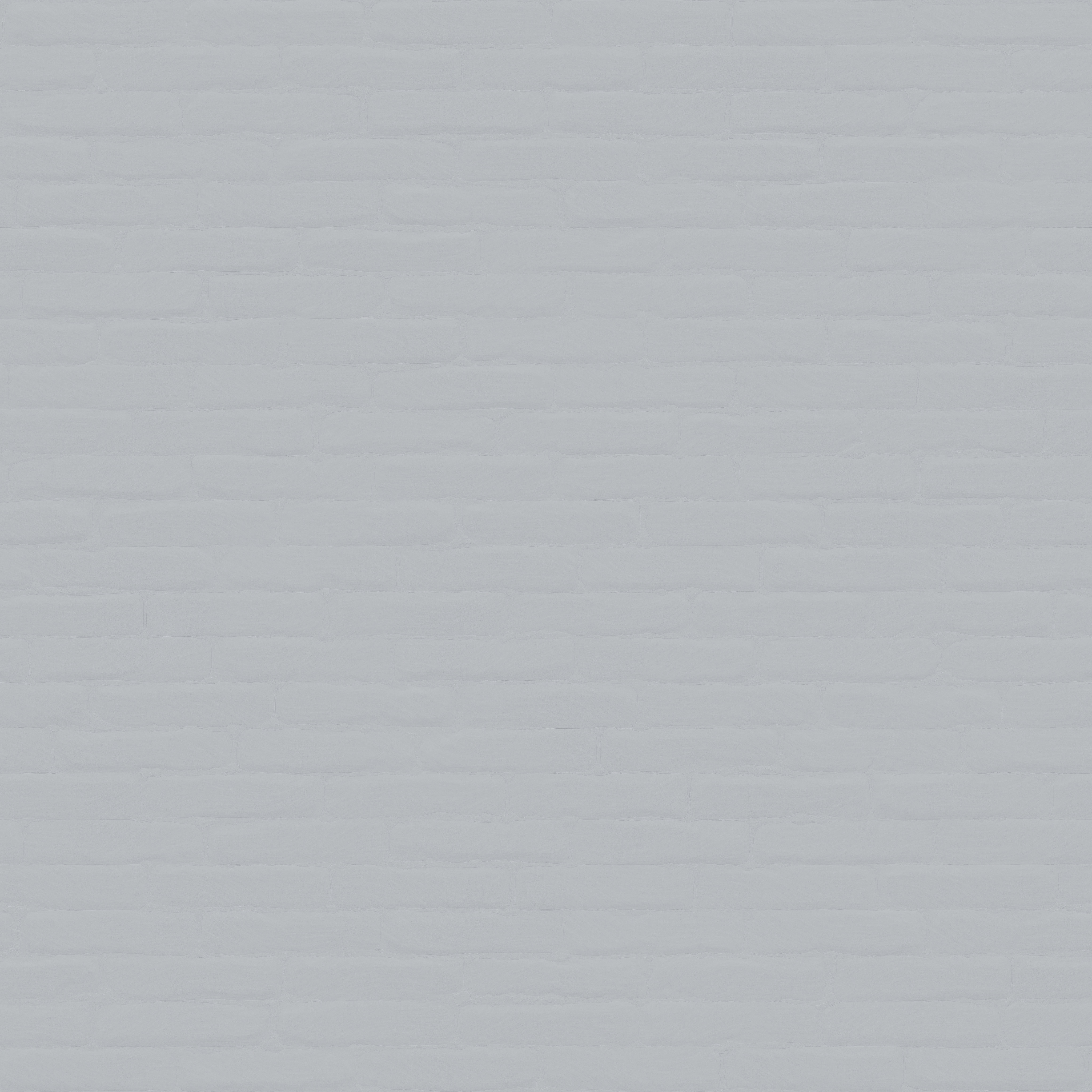

Supplement: Supplementary file 1 — Supplementary file1 (ZIP 85420 KB) [file 426_2026_2297_MOESM1_ESM.zip › SupplementalMaterials/Poliigon_Free_Textures/BricksPaintedWhite001_COL_4K.jpg]

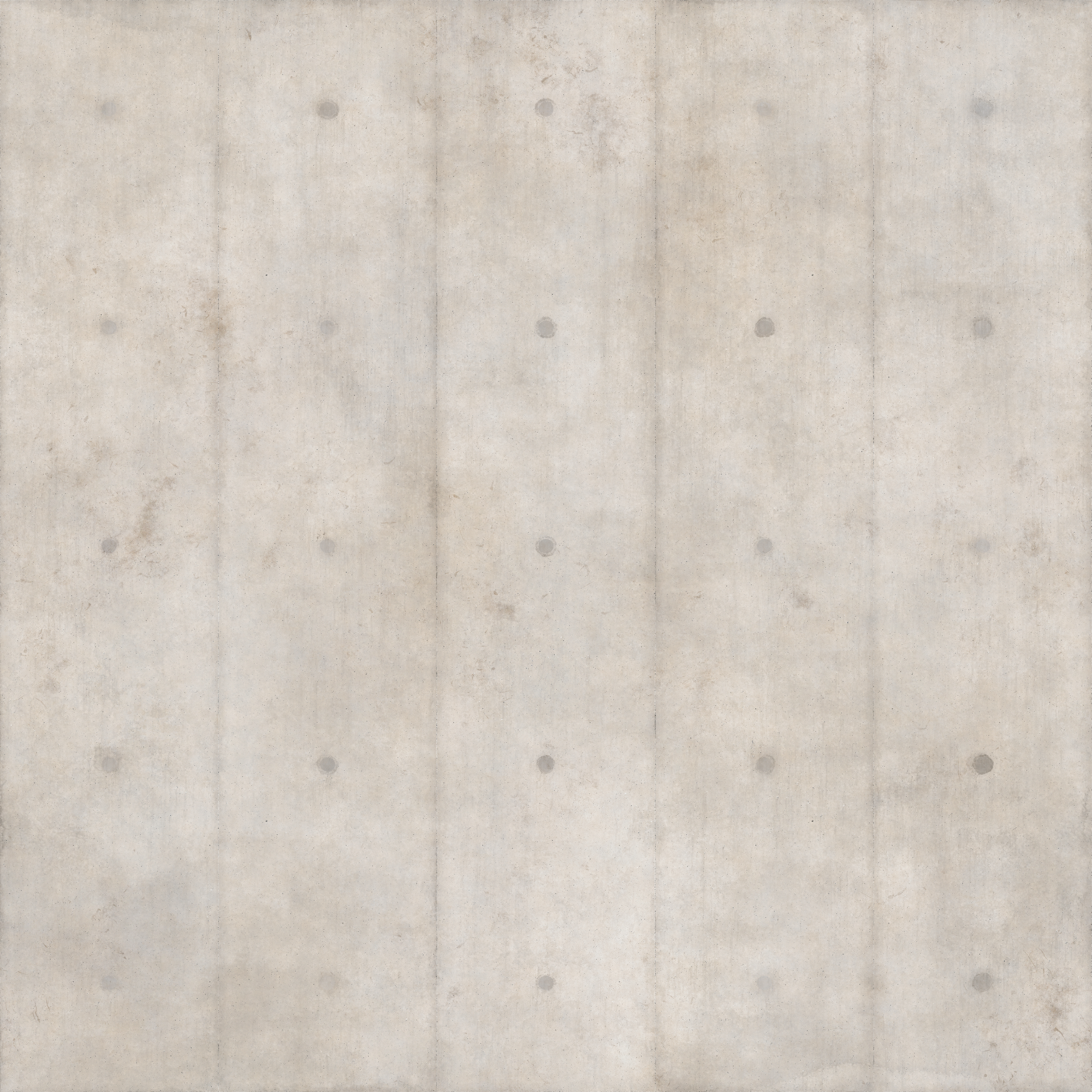

Supplement: Supplementary file 1 — Supplementary file1 (ZIP 85420 KB) [file 426_2026_2297_MOESM1_ESM.zip › SupplementalMaterials/Poliigon_Free_Textures/ConcretePanelsVerticalLarge003_COL_VAR1_4K.jpg]

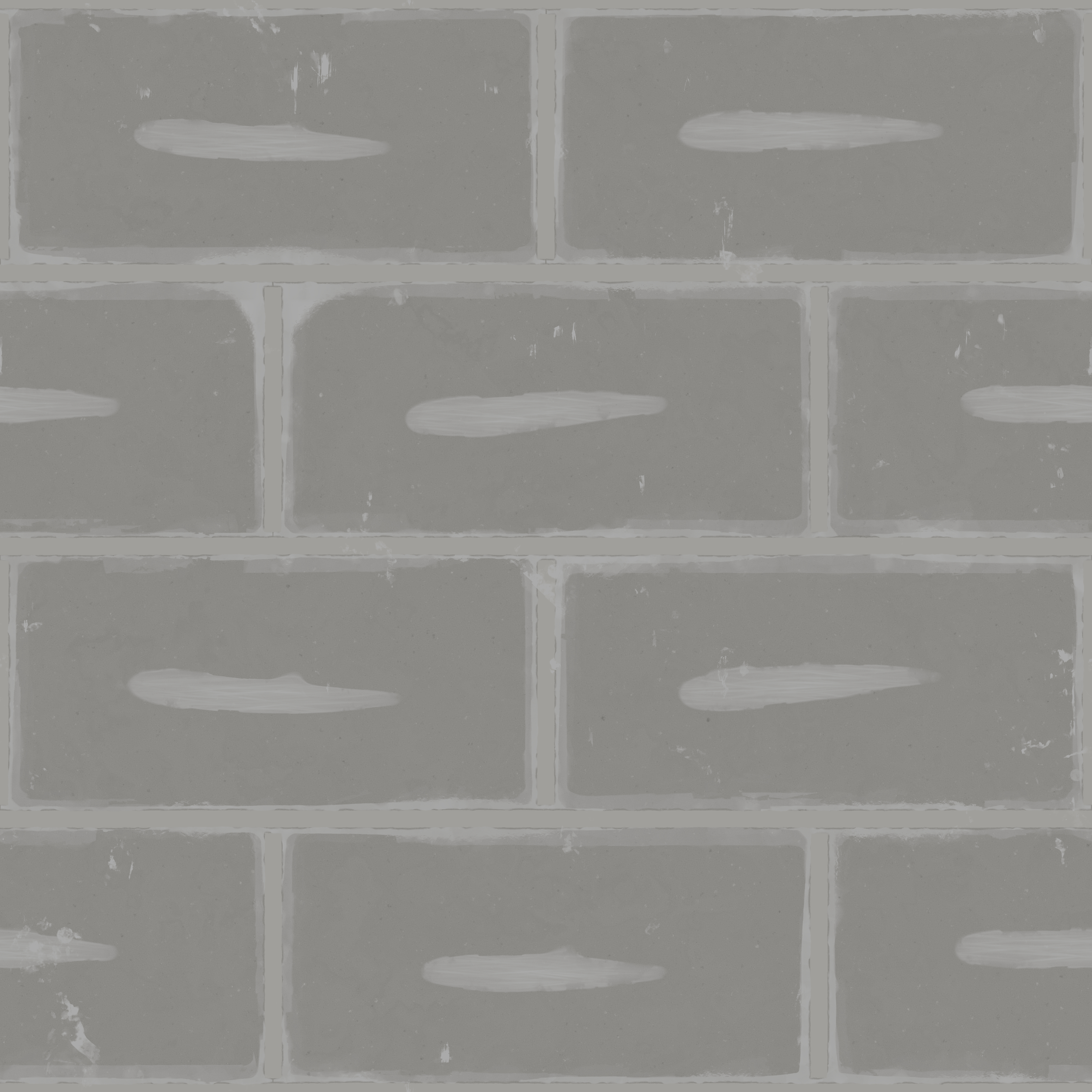

Supplement: Supplementary file 1 — Supplementary file1 (ZIP 85420 KB) [file 426_2026_2297_MOESM1_ESM.zip › SupplementalMaterials/Poliigon_Free_Textures/DrywallPrepared007_COL_VAR1_4K.jpg]

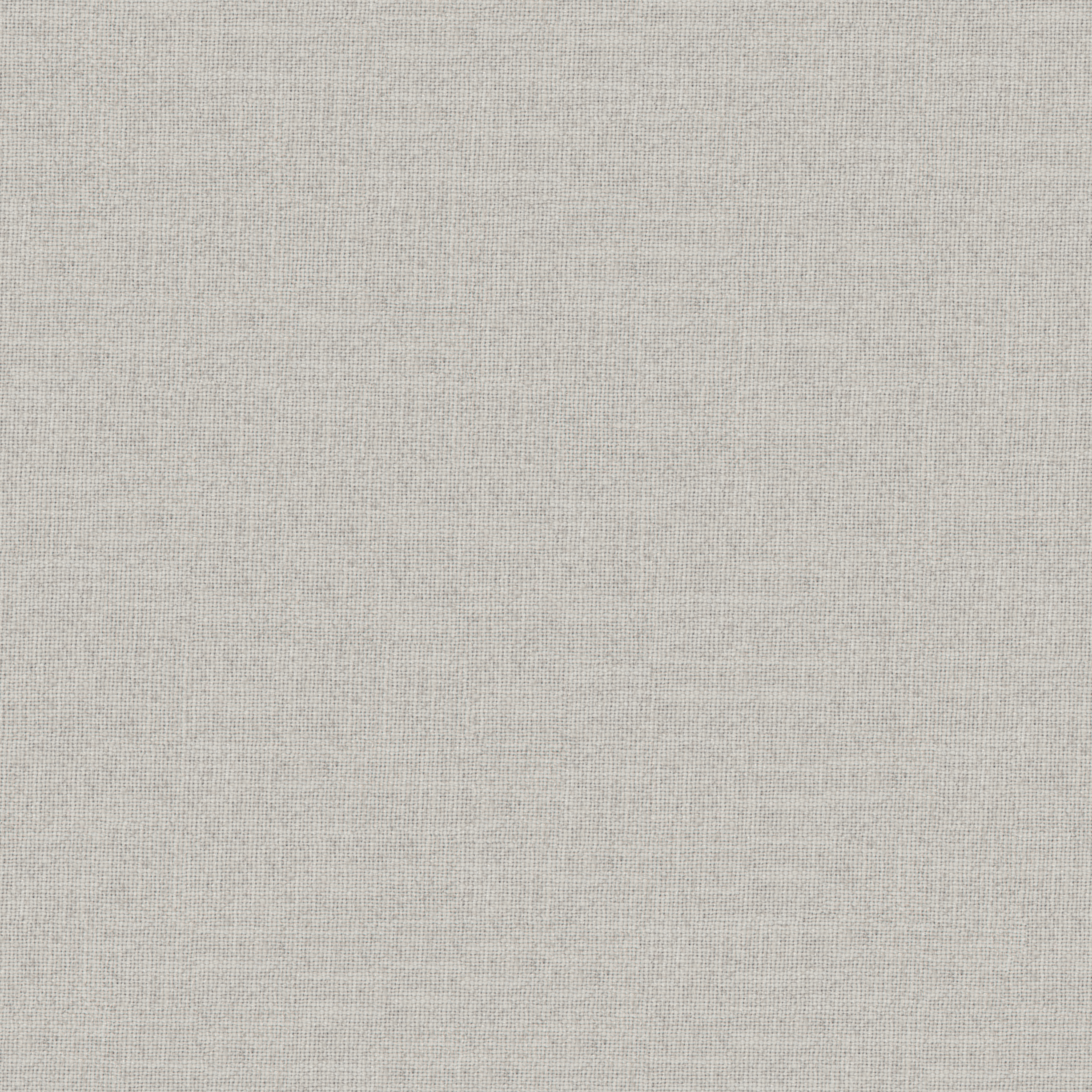

Supplement: Supplementary file 1 — Supplementary file1 (ZIP 85420 KB) [file 426_2026_2297_MOESM1_ESM.zip › SupplementalMaterials/Poliigon_Free_Textures/FabricPlainGreyFlat015_COL_4K.jpg]

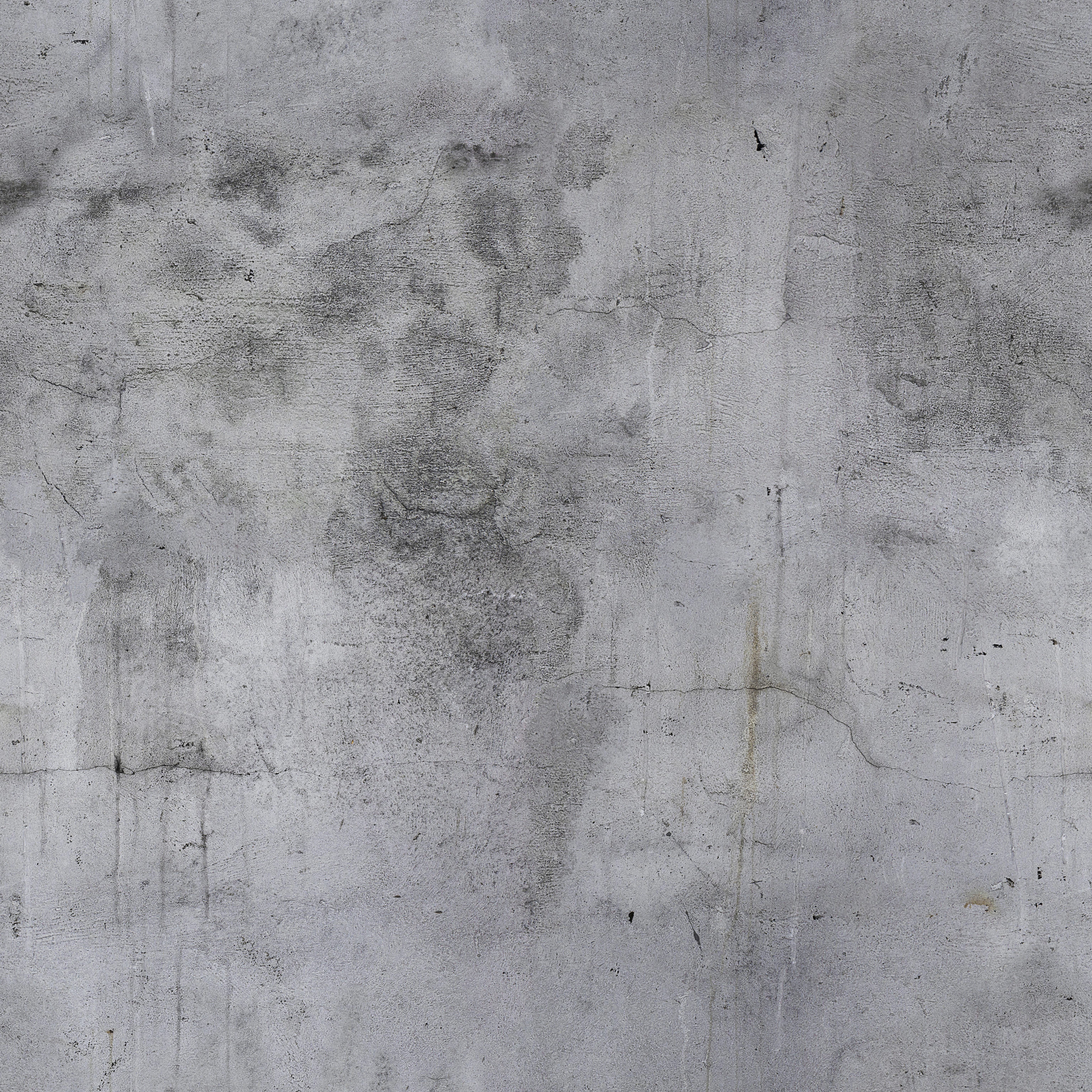

Supplement: Supplementary file 1 — Supplementary file1 (ZIP 85420 KB) [file 426_2026_2297_MOESM1_ESM.zip › SupplementalMaterials/Poliigon_Free_Textures/Plaster17_COL_VAR1_6K.jpg]
